# Seed yield and nutrition in slow-wilting soybean breeding lines as influenced by irrigated and non-irrigated conditions in the Midsouth USA

**DOI:** 10.3389/fpls.2025.1662965

**Published:** 2026-01-06

**Authors:** Nacer Bellaloui, James R. Smith, Alemu Mengistu, Hamed K. Abbas

**Affiliations:** 1 Crop Genetics Research Unit, United States Department of Agriculture (USDA), Agriculture Research Service, Stoneville, MS, United States; 2 Crop Genetics Research Unit, United States Department of Agriculture (USDA), Agriculture Research Service, Jackson, TN, United States; 3 Biological Control of Pests Research Unit, United States Department of Agriculture (USDA), Agriculture Research Service, Stoneville, MS, United States

**Keywords:** soybean, seed composition, seed nutrition, drought tolerance, slow-wilting, seed protein, seed oil

## Abstract

Soybean seed is an important source of protein, oil, and sugars. Soymeal quality is determined by protein and oil contents and sugar profile. Drought results in yield loss and poor seed quality, affecting seed nutrition, including protein, oil, and sugars. Therefore, identifying breeding lines tolerant to drought and investigating possible drought response mechanisms for these lines are important. The objective of this research was to investigate the response of seed yield and seed composition of slow-wilting soybean breeding lines under irrigated and non-irrigated conditions. A 3-year experiment was conducted in Stoneville, MS, USA, in 2015, 2016, and 2018 using slow-wilting (SW) (drought tolerant) trait breeding lines (N98–7265 and PI 471938) of maturity group (MG) V. The checks were 5601T (standard public variety), A5959 (fast-wilting, FW, commercial variety), and Hutcheson (FW parent). The results showed that under irrigated conditions, seed protein was lower and oil was higher in SW genotypes compared with those of checks. No consistent pattern was shown for oleic and linolenic acids under irrigation conditions. Under non-irrigated conditions, seed protein and oleic acid were higher in SW genotypes than checks. Oil content was inconsistent in SW across years. Seed yield was lower in SW genotypes under irrigated and non-irrigated in all 3 years. One possible response mechanism of SW trait is that SW soybean has the ability to maintain leaf water potential and conserve water to use it during drought period. This is reflected by the fact that SW genotypes maintained leaf water potential (LWP) at about -1.20 Ψw under non-irrigated conditions compared with those of checks (about -2.77 Ψw). Moreover, SW genotypes may use high seed sugar (stachyose) accumulation as a response mechanism under non-irrigated conditions to protect seed from damage. The lower yield of SW genotypes cannot be explained by LWP due to their consistent lower yield in all 3 years. This research helps to understand the mechanisms of drought tolerance and helps breeders to select varieties for high seed nutritional qualities such as higher desirable sucrose and lower undesirable raffinose and stachyose under the harsh conditions of high heat and drought of midsouth US.

## 1 Introduction

Soybean is one of the most grown legume in the world, and its seed is a major source of protein and oil (Medic et al., 2014). Soybean seed contains approximately 38% to 40% protein, 18% to 23% oil, and approximately 24% carbohydrates. Higher seed protein, oil, and sucrose are desirable, and higher seed raffinose and stachyose are undesirable. Sucrose contributes to flavor and improves taste in soy-based products such as tofu, soymilk, and natto (Hou et al., 2009). Raffinose and stachyose have negative impacts on the nutritive value of soymeal as they are indigestible by humans and monogastric animals, causing flatulence or diarrhea in nonruminants (Liu, 1997; Hou et al., 2009).

Soybean in USA is grown under irrigated and rain-fed production conditions (Heatherly and Tyler, 1998). However, due to rain fluctuation, especially in Midsouth USA, soybean is subjected to drought, leading to yield loss and poor seed quality (Heatherly and Tyler, 1998; Li et al., 2024), impacting farmers’ economic financial health and profitability. Therefore, developing and growing drought-tolerant soybean varieties under drought and high heat of Midsouth USA is important.

Drought effects on physiological and biochemical pathways in plants have been intensively studied and well documented (Marschner, 2012; Mengel and Kirkby, 1982). Although crop management practices such as irrigation, increasing soil holding field capacity, planting date, and soybean maturity limited yield loss, the most efficient and cost-effective way is breeding selection for drought tolerance (Prasad et al., 2017; Zhang et al., 2019; Bobojonov et al., 2013; Vogel et al., 2021). It was reported that improving soybean drought tolerance using yield only has not been completely successful (Arya et al., 2021; Wei et al., 2018; Yu et al., 2021). This is because of the low heritability of yield under drought conditions and significant interactions between genotype and environment (Ceccarelli et al., 1991). Therefore, developing soybean varieties tolerant to drought has been one of the major goals of breeders (King et al., 2009; Ries et al., 2012; Sinclair et al., 2010; Steketee et al., 2020).

Sinclair et al. (2004) suggested that breeding soybean genotypes based on physiological traits that enhance drought tolerance could be a successful alternative to improve soybean tolerance. It was reported that morpho-physiological traits that contribute to drought tolerance enhancement including carbon isotope ratio (Bai and Purcell, 2018a; Bazzer et al., 2020a, 2020b; Dhanapal et al., 2015; Kaler et al., 2017a, b, 2018a), water use efficiency (WUE) (Blum, 2009; Sinclair, 2012; Vadez et al., 2014), canopy wilting (Chamarthi et al., 2021, Chamarthi et al., 2024; Steketee et al., 2020; Ye et al., 2020), canopy temperature (Bai and Purcell, 2018b; Bazzer and Purcell, 2020; Kaler et al., 2018b), and relative water content of leaves (Babu et al., 2003) may enhance drought tolerance. In the current research, soybean breeding lines with slow wilting (SW) trait were used to investigate the responses of yield and seed nutritional composition (seed protein, oil, fatty acids, and sugars) to drought and high heat conditions of Midsouth USA.

Soybeans tolerant to drought are genotypes that possess a SW trait. Li et al. (2024) reported that SW trait is characterized by a delayed response of the canopy wilting to water deficient in soil compared with the common fast-wilting (FW) soybean cultivars. It was explained that SW soybean conserves soil moisture, but fast-wilting soybean rapidly exhausts water (King et al., 2009; Ries et al., 2012), resulting in the fact that SW soybean maintains or increases yield under drought stress by more than 80% in most soybean-growing regions of the USA (Sinclair et al., 2010). Based on this, it was suggested that SW in soybean could be a promising trait for crop improvement for drought tolerance. Carter et al. (2006) screened exotic germplasm for drought tolerance in North Carolina and identified multiple SW genotypes, for example, PI 416937 and PI471938 (one of the SW lines used in this study). “USDA-N8002” is a soybean cultivar derived from a cross between PI 471938 (25% pedigree) and PI 416937 (12.5% pedigree). This results in a SW cultivar that had yields averaging 7% greater than the cultivar check across 74 environments in southern United States (Carter et al., 2016). The same approach was recently used and resulted in several new genotypes with SW trait (Kaler et al., 2017a; Steketee et al., 2020). It was reported that SW is related to soil moisture conservation and the ability of the plant to use conserved soil water during drying soil period (drought) (King et al., 2009; Ries et al., 2012). On the other hand, SW trait is a complex trait and controlled by several genes (Steketee et al., 2020). Recent physiological studies related to SW were conducted, for example, Chamarthi et al. (2021); Kaler et al. (2017b), and Bazzer et al. (2020a, b) used carbon isotope (^13^C) ratio (as a measure of water use eﬃciency), oxygen isotope (^18^O) ratio (as a measure of transpiration) (Kaler et al., 2017b), canopy temperature (Kaler et al., 2018a, b; Bazzer and Purcell, 2020), and SW characteristics (Chamarthi et al., 2023). However, there is a trade-off between drought tolerance and yield, and this concept was previously reported—for example, Ye et al. (2019) studied the impact of drought on 46 RILs in two population. They found that, under irrigated conditions, the FW RILs had a yield advantage of 7.8%–10.3% compared with the SW RILs in two locations; however, these differences were not statistically significant. Under non-irrigated conditions, however, the FW RILs had lower yield (a reduction of 48.5% and 36.8%) in the two locations. The SW RILs yielded much lower (a reduction of 35.4% and 22.9%) in two locations, showing that the SW RILs had yield advantages of 12.8% and 13.7% compared with the FW lines under non-irrigated conditions in both locations. It is worth noticing that the yield was not significantly different between FW and SW RILs under either conditions when yield was separately evaluated for each RIL population. They explained that the lack of significant difference in yield was due to the reduced sample size. Moreover, Cho et al. (2003) showed that PI 416937 had a low yield potential. Others explained that when water supply is not limiting, a limiting maximum transpiration rate may result in yield penalty (Sinclair et al., 2005).

Genomics and transcriptomes analysis were used to identify QTL associated with SW trait using biparental lines (RILs); molecular markers such as SNPs, using GWAS, differential gene expression, and genes involved in regulations and pathways were involved in drought responses—for example, Li et al. (2024) were able to identify four QTL associated with SW on chromosomes 8, 13, 14, and 17 in RIL population, developed from a cross between KS4895 and Jackson. These QTL accounted for 16%–47% of phenotypic variance in canopy wilting (Charlson et al., 2009; Abdel Haleem et al., 2012). They were able to identify seven QTL in 150 RILs developed from a cross of Benning × PI 416937 and accounted for approximately 75% of observed variation in canopy wilting (Abdel Haleem et al. (2012). Eight QTL clusters containing QTL for canopy wilting were identified (Hwang et al., 2015). Kaler et al. (2017a, b), using GWAS, identified 61 SNPs in a panel of 373 accessions, 45 SNPs in 162 accessions (Steketee et al., 2020), and 188 SNPs in 200 accessions (Chamarthi et al., 2021) on similar genomic regions on chromosomes 1, 4, 6, 9, 12, 15, 18, 19, and 20.

Chamarthi et al. (2021), using GWAS and 200 diverse maturity group (MG) IV accessions and 34,680 SNPs, were able to identify 188 significant SNPs associated with canopy wilting in approximately 152 loci. They identified 183 candidate genes near 188 significant SNPs, and among the 183 candidate genes, 57 SNPs were present within genes coding for proteins with biological functions involved in plant stress responses. Using five biparental mapping populations, Hwang et al. (2015) were able to identify clusters of QTL for SW in at least two populations. A meta-analysis of these eight clusters resulted in identifying nine meta-QTL on eight chromosomal regions (Hwang et al., 2016). Using association mapping, 61 SNPs in a panel of 373 maturity group (MG) IV accessions (Kaler et al., 2017a) and 45 SNPs in a panel of 162 MG VI–VIII accessions (Steketee et al., 2020) were identified. Similar genetic loci regions were identified on Gm01, Gm04, Gm06, Gm09, Gm12, Gm15, Gm18, Gm19, and Gm20 (Hwang et al., 2016). In addition to SW trait, Chamarthi et al. (2021) studied the association of SW loci with loci associated with other drought-tolerant traits such as carbon isotope (^13^C) ratio (as a measure of water use effeciency) (Kaler et al., 2017b; Bazzer et al., 2020a, b), oxygen isotope (^18^O) ratio (as a measure of transpiration) (Kaler et al., 2017b), and canopy temperature (Kaler et al., 2018a, b; Bazzer and Purcell, 2020).

Transcript abundance analysis could be a vital functional genomics tool to examine drought-tolerance-responsive mechanisms and identify genes responsible for drought tolerance. Recently, genome-wide changes associated with gene expression are investigated through microarray chip analysis, RNA-seq approach, and high-coverage gene expression profiling analysis for better understanding of the transcriptional response in relation to water stress. Transcripts were examined in the root tip, including the hypocotyl of soybean, using high-coverage gene expression profiling analysis: 5,831 out of 29,388 were significantly altered under water stress; leaf tissue, 2,724 genes for drought (185); leaf, hypocotyl, and root, 17 proteins (188); roots, 46 proteins (189); and root tips, three S-adenosylmethionine synthetases proteins (190).

Based on the abovementioned literature, although genomic regions and candidate genes for drought traits (canopy slow wilting, water conservation, and low stomatal conductance; delaying of leaf water pressor turgor; water use efficiency, nitrogen fixation; ^13^ C ratio use) (Chamarthi et al., 2022) have been identified, no drought-tolerant cultivars are available in the market due to the complex interactions between drought trait and environment and lack of complete understanding of physiological and genetic mechanisms associated with the trait due to the reason cited above. Therefore, developing drought tolerance (SW) soybean across locations and years remains a challenge, and further studies are needed to understand the physiological and genetic mechanisms of drought. Thus, the objective of this research is to investigate the response of yield and seed nutrition (seed protein, oil, fatty acids, and sugars) in breeding lines with SW trait under high heat and drought conditions of the midsouth USA.

## 2 Materials and methods

A repeated field experiment was conducted at the Jamie Whitten Delta States Research Center in Stoneville, MS, USA. SW PI 471938 (parent) and N98-7265 (progeny) were used (seeds were obtained from Dr. Jimmy Carter, USDA-ARS, Raleigh, NC, USA), and the seeds were increased at Stoneville, MS, USA, in 2013 and 2014. Field experiments were conducted in 2015, 2017, and 2018 (2016 was skipped due to its wet growing season during planting). N98–7265 was a cross between Hutcheson (FW parent) and PI 471938 (SW parent). The checks were 5601T (standard public variety) and A5959 (FW commercial variety). Harvesting was conducted using a two-row field combine harvester. The experiment was irrigated and non-irrigated, and the design was a split plot, with irrigation as the main plot and genotype as the sub-plot (**Supplementary Figure S1**). Watermark 200SS soil sensors (Irrometer Company, Inc., Riverside, CA, USA) were inserted into the soil to measure soil water potential in irrigated and non-irrigated plots. Well-watered plants were kept between -15 and - 25 kPa (field capacity level), and non-irrigated plots showed a soil potential between -90 and -180 kPa. A furrow irrigation was used to water irrigated plots. The plots were monitored regularly with tensiometer readers, and we irrigated the irrigated plots for the irrigated treatments whenever the tensiometers read between -90 and -180 kPa or between -150 and -180 kPa (timing of irrigation/days of irrigation and the amount of irrigation water in each year are shown in **Supplementary Table S1**; irrigation times and amounts are shown in **Supplementary Table S1**). The seeding rate was 25 seeds per meter of row, the row spacing was 0.91 m, and the row length was 5.79 m long at planting but end-trimmed to 4.88 m after R5. Seeds were harvested when they reached R8 (full maturity, 95% of pods reached full maturity) according to Fehr and Caviness (1977).

### 2.1 Leaf water potential measurement

Leaf water potential (Ψw) was determined on young and fully expanded leaves at vegetative stage using leaf cutter thermocouple psychrometers (J.R.D. Merrill Specialty Equipment, Logan, UT, USA) at mid-day (1200–1300 h). A 5-mm diameter leaf disc was taken by using a cork borer and placed in a leaf cutter thermocouple psychrometer. Leaf water potential measurements were conducted on three individual plants (replicate) of each genotype from each plot under irrigated and non-irrigated conditions. The leaf cutter thermocouple psychrometers were placed in a water bath at 25°C for 4 h. Outputs from the psychrometers were recorded by a PSYPRO data logger (WESCOR, Inc., Logan, UT, USA).

### 2.2 Seed protein, oil, fatty acids, and sugars

Mature seeds were collected at R8, and seed composition components [seed protein, oil, fatty acids, and sugars (sucrose, raffinose, and stachyose)] were analyzed. Seed composition constituents were analyzed with a Diode Array Feed Analyzer AD 7200 (Perten, Springfield, IL, USA). Briefly, seeds were ground by using a Laboratory Mill 3600 (Perten), and approximately 25 g of ground seed was analyzed for seed composition components as described elsewhere (Bellaloui et al., 2009, 2009; Bellaloui et al., 2014; Wilcox and Shibles, 2001). Calibration equations were initially developed by the University of Minnesota and upgraded by the Perten company, using Perten’s Thermo Galactic Grams PLS IQ software. The calibration equations were established using standard laboratory protocols according to AOAC methods (AOAC, 1990a, b). Protein, oil, and sugars (sucrose, raffinose, and stachyose) were expressed on a dry matter basis (Piper and Boote, 1999; Bellaloui et al., 2009; Maestri et al., 1998), while fatty acids palmitic, stearic, oleic, linoleic, and linolenic were expressed on a total oil basis.

### 2.3 Experimental design and statistical analysis

The experimental design was a randomized complete block design with a split plot with irrigation (treatment) as a main plot and genotype (line) as a sub-plot. Our study was somewhat unique in that we actually replicated the irrigation treatment—that is, we did not just have replications within a drought block and an irrigation block (which does not replicate the irrigation treatment), but we actually replicated the irrigation treatment, having four total blocks with irrigation assigned to two blocks and non-irrigation assigned to two blocks. Analysis of variance using Proc Glimmix was conducted, using SAS (SAS, 2013). The model used was: *yijkl* = *u* + *Yi* + *Cj* + *Tk* + *rl(i*) + (*YC*)*ij* + (*YT*)*ik* + (*CT*)*jk* + (*YCT*)*ijk* + *eijkl*, where _yijkl_ = observed phenotype of genotype j in year *i*, treatment k, replicate *l*; *u* = overall mean; *Yi* = fixed effect of year *i*; C*j* = fixed effect of genotype *j*; *Tk* = fixed effect of treatment k; rl_(i)_ = random effect of replicate *l* nested within year *i*; (*YC*)*ij* = interaction effect between year *i* and genotype *j*; (*YT*)*ik* = interaction effect between year *i* and treatment *k*; (*CT*)*jk* = interaction effect between genotype *j* and treatment *k*; (*YCT*)*ijk*: interaction effect between year *i*, genotype *j*, and treatment *k*; *eijkl* = residual error. Means were separated by using Fisher’s least significant difference test at the 5% probability level. Correlation was conducted using SAS. Means were separated using Fisher’s protected least significant difference (LSD) at 0.05 level of significance. PROC CORR in SAS was used to establish correlations (*R* and *P*-values).

## 3 Results and discussion

**Figure 1:**
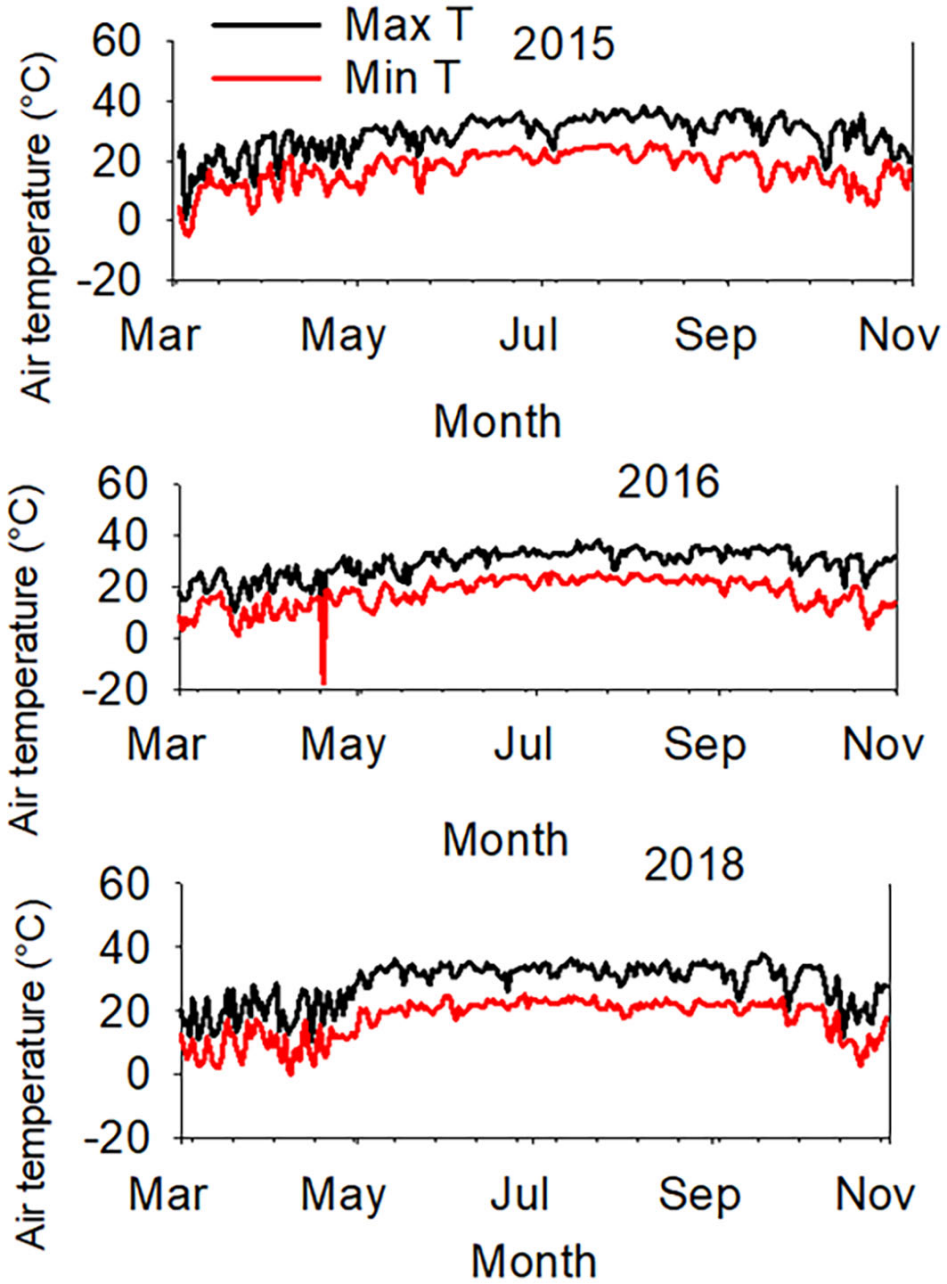
Daily weather components (maximum air temperature (°C), Max Temp, and minimum air temp (°C), Min Temp, in years 2015 (top), 2016 (middle), and 2018 (bottom).

**Figure 2:**
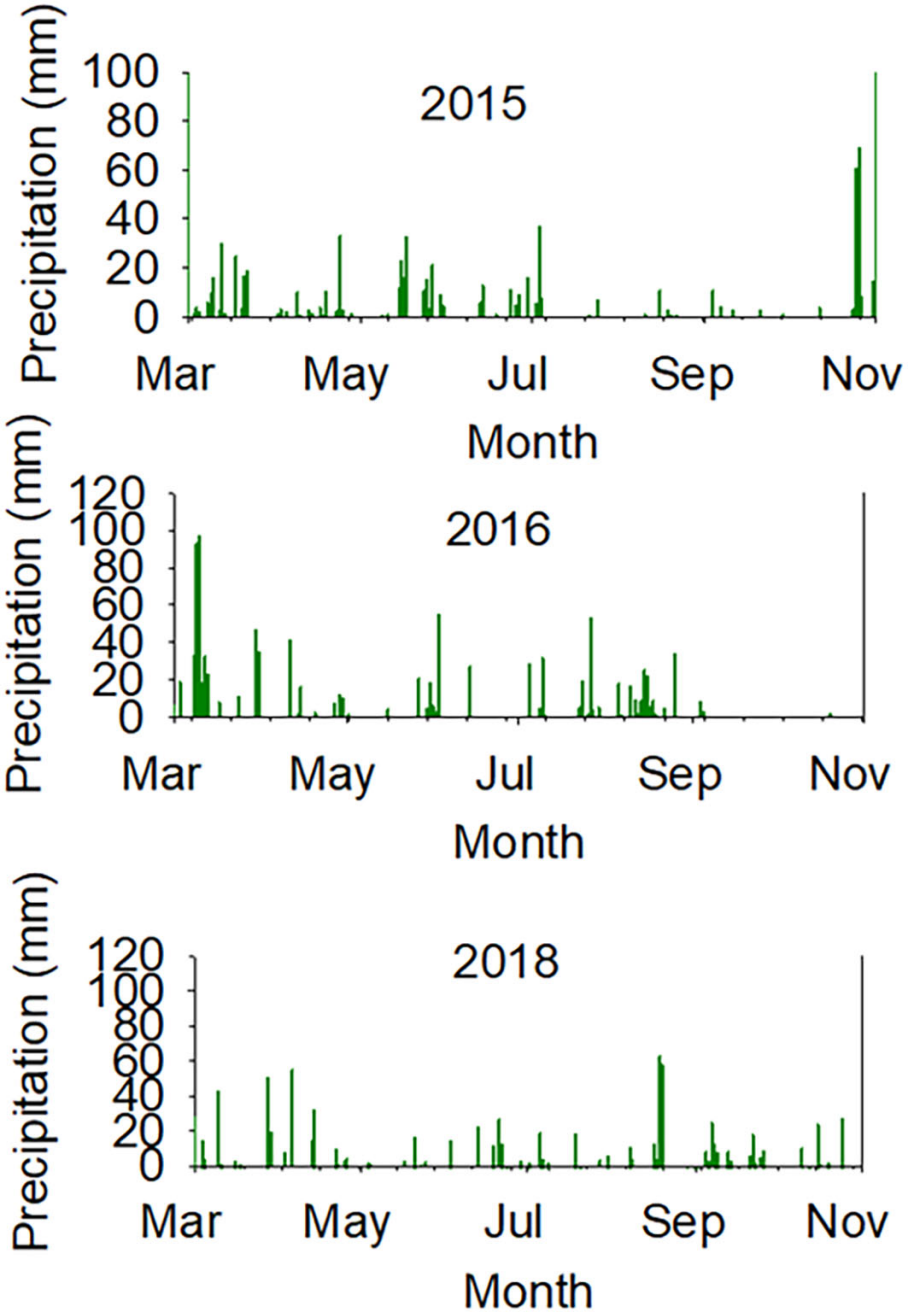
Daily weather components (precipitation, mm) in years 2015 (top), 2016 (middle), and 2018 (bottom).

ANOVA (**Tables 1**, **2**) show that line had a significant effect on oil, oleic acid, sucrose, stachyose, and yield. Irrigation treatment (Treat) had significant effects on protein, oleic, sucrose, stachyose, and yield. Except for raffinose and stachyose, Line × Year interactions had significant effects for all seed composition components and yield (**Tables 1**, **2**) due to the weather components (air temperature and precipitation in each month and daily) differences (**Table 3**; **Figures 1**, **2**). Experiment (Exp) had no significant influence on seed composition components. A similar observation was noticed for Exp and its interactions with Line or Year for protein, oil, oleic, linolenic, raffinose, stachyose, and yield (**Tables 1**, **2**).

**Table 1:** ANOVA for the effects of year, line, treatment (Treat), experiment (Exp), and their interactions for seed protein, oil, and fatty acids.

Effect	Protein (g/kg)	*P*	Oil (g/kg)	*P*	Oleic acid (g/kg)	*P*	Linolenic acid (g/kg)	*P*
	*F*-value		*F*-value		*F*-value		*F*-value	
Year	1.25	ns	28.47	ns	7.55	***	61.9	***
Line	2.1	ns	9.3	***	33.72	***	1.99	ns
Year * Line	2.51	*	8.27	***	2.59	**	2.93	**
Treat	32.75	***	0.22	ns	32.76	***	0.24	ns
Year * Treat	2.43	ns	5.21	*	11.17	***	4.66	**
Line * Treat	57.34	***	3.47	**	22.82	***	0.26	ns
Year * Line * Treat	0.98	ns	4.02	***	1.25	ns	0.6	ns
Exp	0.01	ns	2.89	ns	0.00	ns	0.06	ns
Year * Exp	2.95	ns	0.38	ns	1.57	ns	1.16	ns
Line * Exp	2.27	ns	0.29	ns	1.51	ns	2.21	ns
Year * Line * Exp	1.52	ns	0.6	ns	0.6	ns	0.57	ns
Treat * Exp	1.55	ns	2.15	ns	0.69	ns	0.51	ns
Year * Treat * Exp	0.41	ns	1.68	ns	0.61	ns	0.05	ns
Line * Treat * Exp	0.85	ns	0.73	ns	1.72	ns	0.48	ns
Year * Line * Treat * Exp	0.71	ns	0.61	ns	0.81	ns	0.75	ns
Residual	73.33		60.64		300		175	

The experiment was conducted in 2015, 2016, and 2018 in Stoneville, MS, USA.

*P ≤ 0.05; **P ≤ 0.01; ***P ≤ 0.001.

**Table 2:** ANOVA for the effects of year, line, treatment (Treat), experiment (Exp), and their interactions for seed sucrose, raffinose, stachyose, and seed yield.

Effect	Sucrose (mg/g)		Raffinose (mg/g)		Stachyose (mg/g)		Yield (kg/ha)	
	*F*-value	*P*	*F*-value	*P*	*F*-value	*P*	*F*-value	*P*
Year	3.94	ns	19.44	ns	147	***	16.91	***
Line	4.6	**	0.84	ns	23.88	***	170	***
Year * Line	6.12	***	1.08	ns	1.29	ns	7.12	***
Treat	8.27	**	3.14	ns	7.63	**	330	***
Year * Treat	4.15	*	0.16	ns	5.51	**	130	***
Line * Treat	3.69	**	2.3	ns	34.15	***	4.12	**
Year * Line * Treat	6.53	***	0.67	ns	1.31	ns	1.1	ns
Exp	1.8	ns	3.59	ns	0.00	ns	1.41	ns
Year * Exp	0.33	ns	4.24	*	1.66	ns	1.57	ns
Line * Exp	1.14	ns	0.36	ns	0.27	ns	0.59	ns
Year * Line * Exp	0.79	ns	0.4	ns	0.44	ns	1.31	ns
Treat * Exp	3.4	ns	0.59	ns	3.13	ns	0.49	ns
Year * Treat * Exp	4.27	**	0.46	ns	0.25	ns	1.15	ns
Line * Treat * Exp	1.14	ns	0.57	ns	0.97	ns	0.84	ns
Year * Line * Treat * Exp	1.71	ns	0.64	ns	1.03	ns	0.12	ns
Residual	14.99		0.14		12.65	***	71,646	

The experiment was conducted in 2015, 2016, and 2018 in Stoneville, MS, USA.

*P ≤ 0.05; **P ≤ 0.01; ***P ≤ 0.001.

**Table 3:** Weather components (maximum air temperature, Max Temp; minimum air temp, Min Temp; and precipitation) in years 2015, 2016, and 2018.

Month	2015 Max temp (°C)	Min temp (°C)	Precipitation (mm)	2016 Max temp (°C)	Min temp (°C)	Precipitation (mm)	2018 Max temp (°C)	Min temp (°C)	Precipitation (mm)
March	18.37	8.31	4.68	20.45	10.02	13.90	19.05	9.05	5.62
April	24.28	14.11	2.53	22.96	12.61	3.21	21.31	9.37	4.56
May	28.66	17.78	4.83	28.15	16.45	1.71	32.08	19.91	1.05
June	32.35	21.28	2.04	32.26	21.93	3.15	32.78	21.91	3.36
July	33.39	23.24	1.85	33.66	23.12	5.15	32.97	22.54	2.18
August	33.49	20.88	0.53	33.08	23.05	5.36	32.85	21.36	5.10
September	32.52	18.20	0.72	33.19	19.56	0.45	31.43	20.96	4.11
October	26.29	13.19	5.15	29.23	13.10	0.12	25.48	14.01	2.33

**Figure 3:**
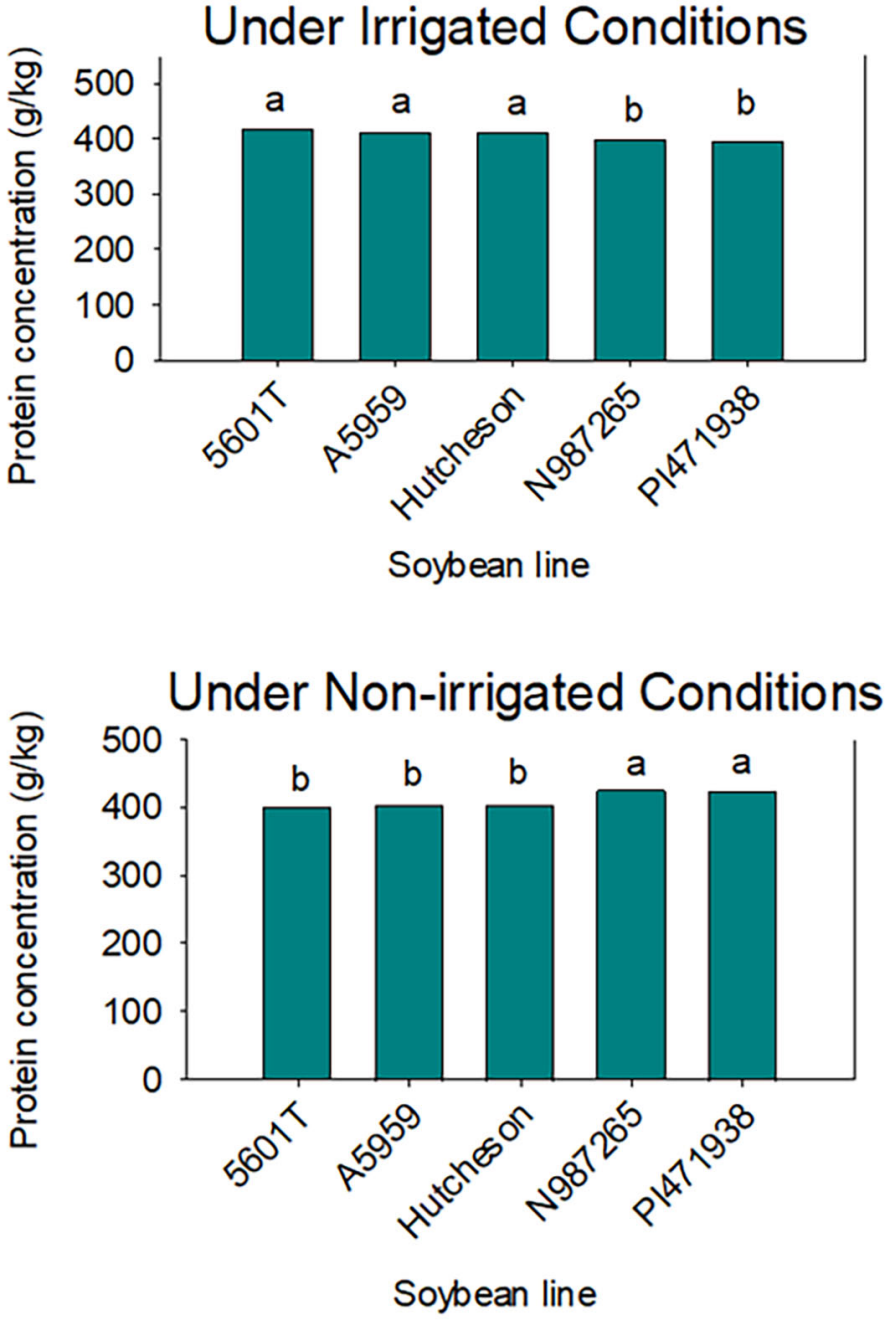
Seed protein in slow-wilting breeding lines (N987265 and PI471938) and the rest of the genotypes (checks). PI471938 is a parent. The data are cross-years (2015, 2016, and 2018) under irrigated (top) and non-irrigated (bottom). Seed protein concentration is higher in checks compared with those of SW lines. The experiment was conducted in Stoneville, MS, USA. Different letters indicate significant differences at P≤0.05; same letters indicate that there was no significant differences at P≤0.05.

**Figure 4:**
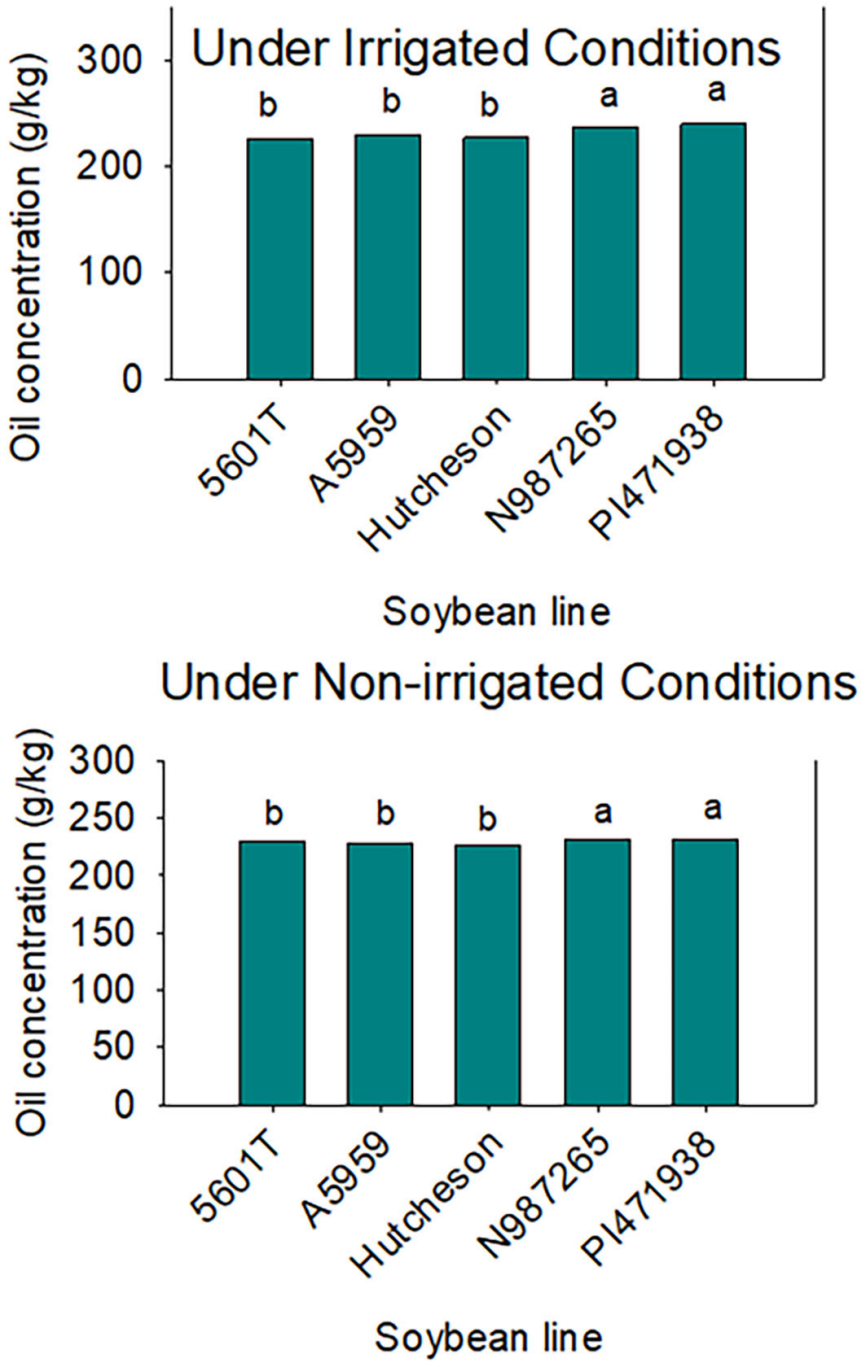
Seed oil concentration in slow-wilting breeding lines (N987265 and PI471938) and the rest of the genotypes (checks). PI471938 is a parent. The data are cross-years (2015, 2016, and 2018) under irrigated (top) and non-irrigated (bottom). Seed oil concentration is higher in SW lines compared with those of checks. The experiment was conducted in Stoneville, MS, USA. Different letters indicate significant differences at P≤0.05; same letters indicate that there was no significant differences at P≤0.05.

**Figure 5:**
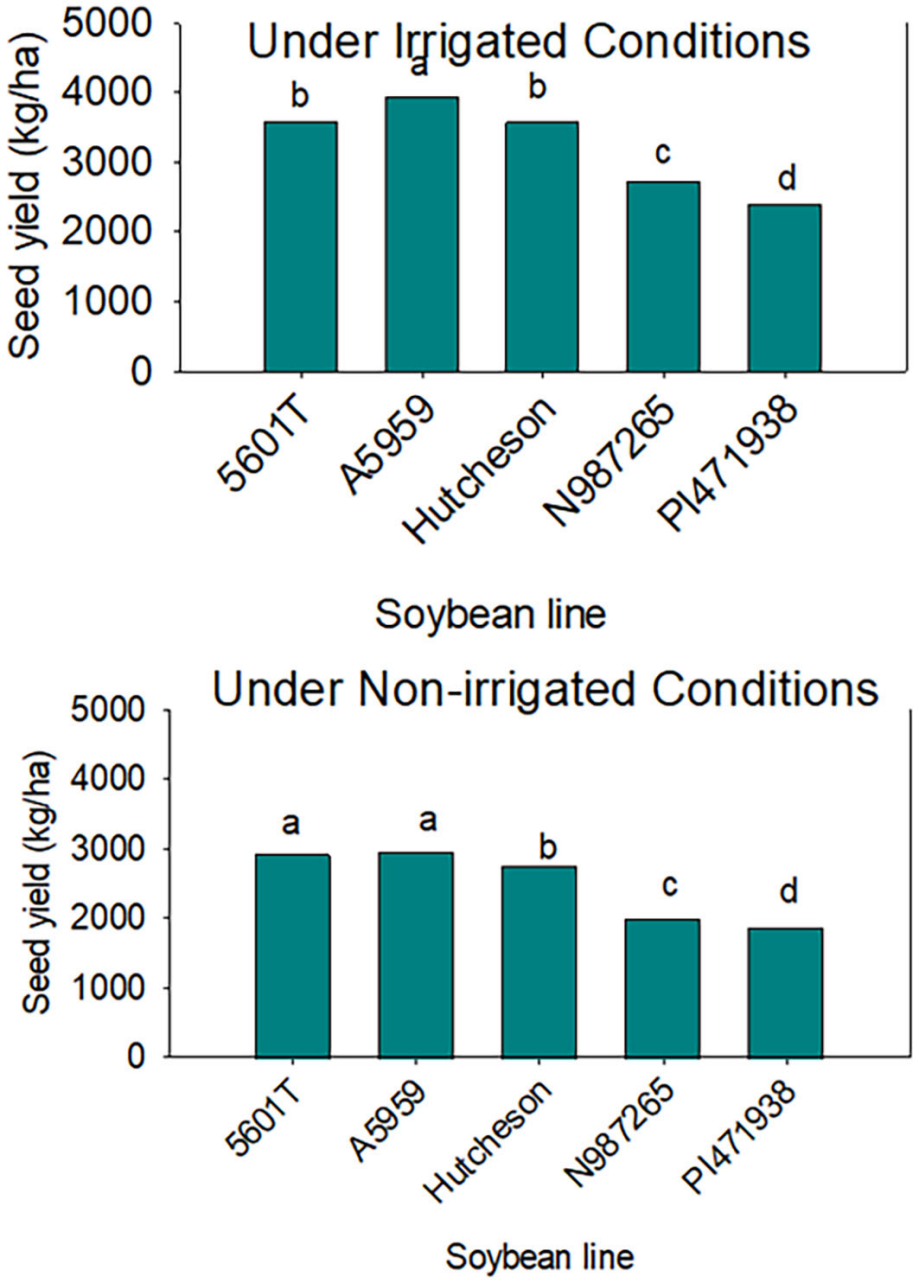
Seed yield in slow-wilting breeding lines (N987265 and PI471938) and the rest of the genotypes (checks). PI471938 is a parent. The data are cross-years (2015, 2016, and 2018) under irrigated (top) and non-irrigated (bottom). Yield is lower in SW lines compared with those of checks. The experiment was conducted in Stoneville, MS, USA. Different letters indicate significant differences at P≤0.05; same letters indicate that there was no significant differences at P≤0.05.

Under irrigation conditions, the mean value of protein in SW genotypes was lower than those of checks, including the parent Hutcheson (**Table 4**). This was shown across years as well (**Figure 3**). However, oil was higher in SW genotypes than the check and the parent Hutcheson, and this was shown across years (**Figure 4**). No consistent pattern was shown for oleic and linolenic under irrigation conditions. This inconsistency of oleic and linolenic acid under irrigated conditions could be due to, at least, two possible reasons. The first reason could be due to the treatment (irrigation) as under-irrigated conditions; the slow wilting trait was not expressed enough to result in a strong consistent response of oleic and linolenic acids. Under irrigation conditions, oleic and linolenic acids may have been driven by the weather components of each year, especially temperature as temperature patterns vary in each year as shown in the weather data. The change in oleic response will lead to changes in linolenic acid as oleic and linolenic acids are negatively correlated. Yield was consistently lower in SW genotypes under irrigated and non-irrigated in each year and cross-years (**Table 4**, **Figure 5**). Under non-irrigated conditions, the mean value of protein and oleic acid was higher in SW genotypes than the checks (**Table 4**). Yield was lower in SW genotypes than the checks. No consistent pattern was shown for sucrose or raffinose; however, stachyose in SW genotypes was higher under non-irrigated conditions in each year than the checks (**Table 5**).

**Table 4:** Mean values for seed protein, oil, fatty acid concentrations (g/kg) (oleic and linolenic acids), and yield (kg/ha) under irrigated and non-irrigated conditions in soybean slow-wilting (SW) breeding lines (N987265 and PI471938) and fast-wilting (FW) genotypes (checks).

Year/line	Irrigated					Non-irrigated				
	Protein	Oil	Oleic	Linolenic	Yield	Protein	Oil	Oleic	Linolenic	Yield
2015										
5601T	419	207	185	61.32	3,574	404	208	177	61.23	2,152
A5959	407	208	166	65.30	3,612	403	206	182	59.28	1,887
Hutcheson	406	212	163	69.87	3,619	403	192	173	63.18	1,797
N987265	391	223	170	60.00	2,996	432	221	225	44.05	1,233
PI471938	384	228	193	51.22	2,691	421	224	250	32.57	1,293
LSD	2.48	2.32	7.02	4.44	115	3.60	5.77	6.36	4.36	56.13
2016										
5601T	420	229	182	75.52	3,648	402	240	162	76.57	3,384
A5959	408	242	178	73.55	3,962	402	246	163	76.87	3,643
Hutcheson	416	232	172	70.42	3,732	411	242	163	72.18	3,573
N987265	400	240	193	67.30	2,673	423	232	205	75.23	2,594
PI471938	395	245	178	71.30	2,339	429	230	199	75.70	2,367
LSD	3.46	3.67	8.35	5.78	111	3.13	2.73	4.98	5.06	156
2018										
5601T	411	239	198	75.30	3,485	399	245	190	80.85	3,248
A5959	411	237	179	85.45	4,226	405	238	169	81.20	3,342
Hutcheson	407	237	172	82.95	3,351	401	250	170	85.57	2,901
N987265	399	247	182	84.18	2,493	424	244	236	91.83	2,232
PI471938	401	245	173	82.07	2,104	426	246	241	94.93	1,991
LSD	3.79	2.72	8.03	5.55	74.72	4.29	2.96	5.55	5.44	89.91

PI471938 is a slow-wilting line parent. N987265 is a cross between PI471938 and Hutcheson (fast-wilting parent). The experiment was conducted in 2015, 2016, and 2018 in Stoneville, MS, USA.

The means in each column were separated by Fisher’s least significant difference test at the 5% probability level.

LSD, least significant difference for each column within each year for each variable.

**Table 5:** Mean values for seed sucrose, raffinose, and stachyose concentrations (mg/g) under irrigated and non-irrigated concentrations.

Year/line	Irrigated			Non-irrigated		
	Sucrose	Raffinose	Stachyose	Sucrose	Raffinose	Stachyose
2015						
5601T	27.23	6.07	26.83	29.12	5.90	23.03
A5959	29.88	6.03	27.70	31.48	6.23	25.93
Hutcheson	31.82	6.03	29.20	29.38	6.22	22.05
N987265	30.93	6.17	27.08	29.00	6.32	37.58
PI471938	30.48	6.10	22.58	30.42	6.22	35.52
LSD	2.20	0.12	1.49	1.72	0.11	1.68
2016						
5601T	30.53	6.03	37.43	35.95	6.00	34.73
A5959	34.87	5.70	39.80	35.42	6.15	34.37
Hutcheson	32.90	6.02	39.50	32.70	6.10	32.08
N987265	33.30	5.87	38.45	34.17	6.03	43.73
PI471938	42.67	6.17	39.10	32.68	6.18	44.62
LSD	1.14	0.13	1.15	1.12	0.12	1.19
2018						
5601T	33.62	7.27	34.42	29.35	7.12	32.87
A5959	29.25	6.80	33.42	28.35	7.05	32.08
Hutcheson	34.20	6.98	34.78	32.58	7.47	34.02
N987265	29.10	6.98	32.83	44.53	7.05	44.17
PI471938	34.57	7.22	36.03	45.12	6.87	44.35
LSD	1.74	0.21	1.16	1.51	0.15	1.48

Means in each column were separated by Fisher’s least significant difference test at the 5% probability level.

LSD, least significant difference for each column within each year for each variable.

It appears that Line, Treat, and Line × Year interactions were important factors influencing the seed composition, especially oil, oleic acid, and sucrose. It is clear that Exp did not have a significant effect on the seed composition component, indicating that the response of these component was the same in each year. This means that the ranking of each component in each year was the same, supported by the fact that Exp × Year was not significant for most of these components (protein, oil, oleic, linolenic, sucrose, stachyose, or yield). The lower seed protein concentrations in SW than the checks under irrigated conditions may indicate the lower efficiency of nutrient uptake, including nitrogen in SW genotypes under irrigation conditions. The higher oil concentration in SW genotypes under irrigated conditions reflects the inverse relationship between protein and oil. The inverse relationship between protein and oil is genetically controlled, and mechanisms controlling this relationship are not completely understood, although it was reported to be associated with carbon and nitrogen metabolism and energy distributions (Ray et al., 2006; Wang et al., 2019; Clemente and Cahoon, 2009). The changes of seed protein and oil content are influenced by the expression of oil and protein genes (Severin et al., 2010)—for example, fad2, lox, and kcs genes involved in fatty acids synthesis; transcription factors such as abi3 and lec2; and sugar transporter SWEET10 involved in both protein and oil (Wang et al., 2020; Peng et al., 2021). Comparing high-protein and high-11S soybeans with their low-protein and low-11S counterparts, it was found that lipid and carbohydrate metabolism were differentially expressed, indicating the involvement of carbon and nitrogen metabolism in seed protein and oil accumulation (Hooker et al., 2022; 2023). Generally, to have a high protein accumulation in the seed, the plant has to direct its energy toward efficient nitrogen uptake (nitrate and/or ammonium) and assimilation to synthesize amino acids through the nitrogen assimilation process and, consequently, protein production. The activity of this complex apparatus depends on the signaling machinery that drives the activity of metabolism and biosynthesis, and this depends on genotype, growing conditions/environment of plants, soil conditions, and nutrient availability in the soil. In case of high accumulation of oil in the seeds, the plant needs to shift its signaling apparatus and plant energy toward sugars/carbohydrates and lipid synthesis. Therefore, the inverse relationship between protein and oil is controlled genetically, but it also is influenced by the signaling apparatus and gene expression that is heavily influenced by the environment. Advanced tools in genomics and transcriptomics will allow us to unfold a lot of functional genomics to understand carbon–nitrogen metabolism and their ratios and understand the major factors involved in the pathways of protein and oil accumulation, especially for drought-tolerant genotypes. This approach may allow us to further understand the inverse relationship between protein and oil and their complex pathways of nitrogen and amino acid, lipid and fatty acid, and sugar biosynthesis.

**Figure 6:**
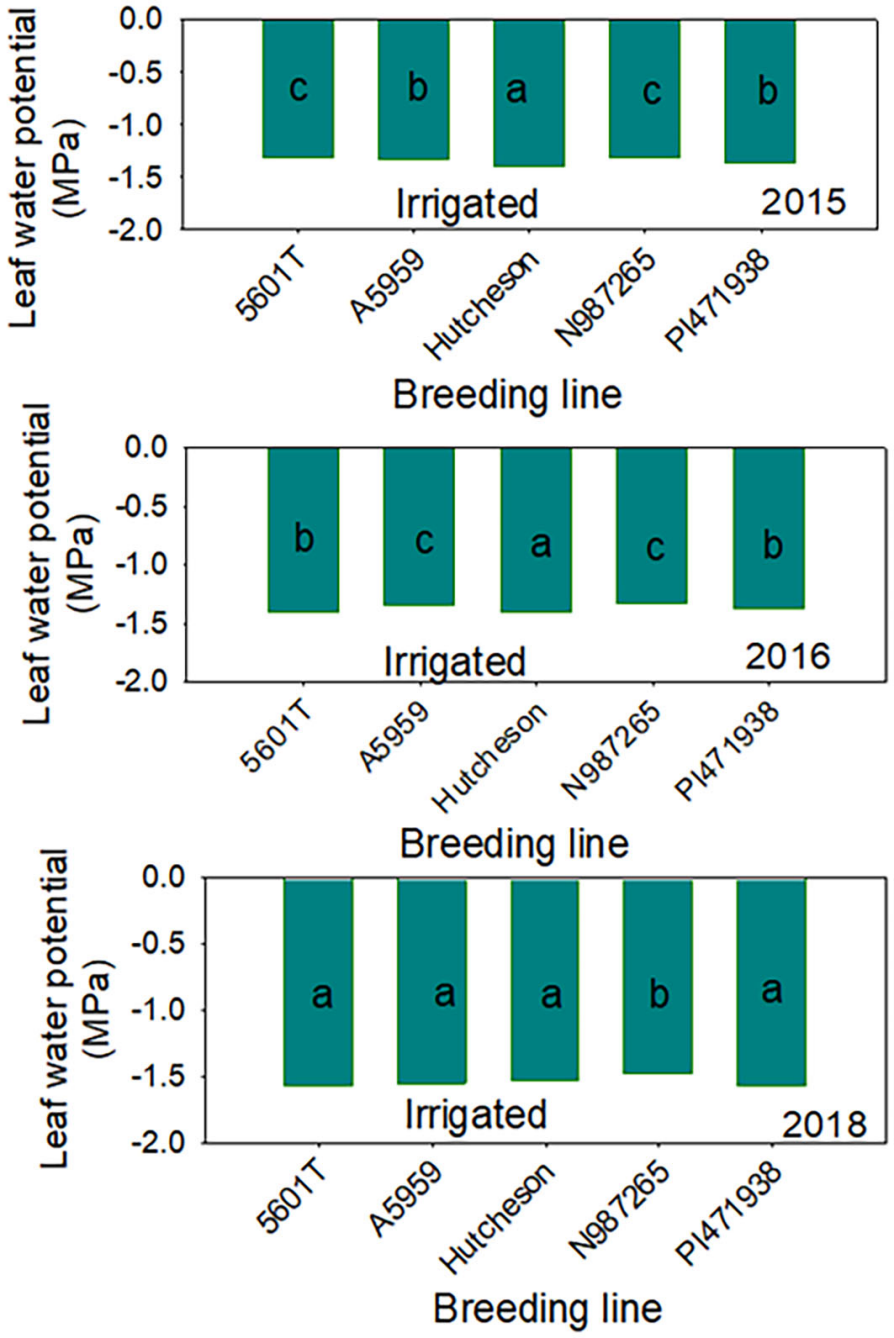
Leaf water potential (Ψw) under irrigated conditions in slow-wilting (SW) (N987265 and PI471938) and fast-wilting (FW) breeding lines/genotypes. Hutcheson, FW parent; PI471938, SW parent; N987265, SW progeny. FW, fast-wilting; SW, slow-wilting. Different letters indicate significant differences at P≤0.05; same letters indicate that there was no significant differences at P≤0.05.

**Figure 7:**
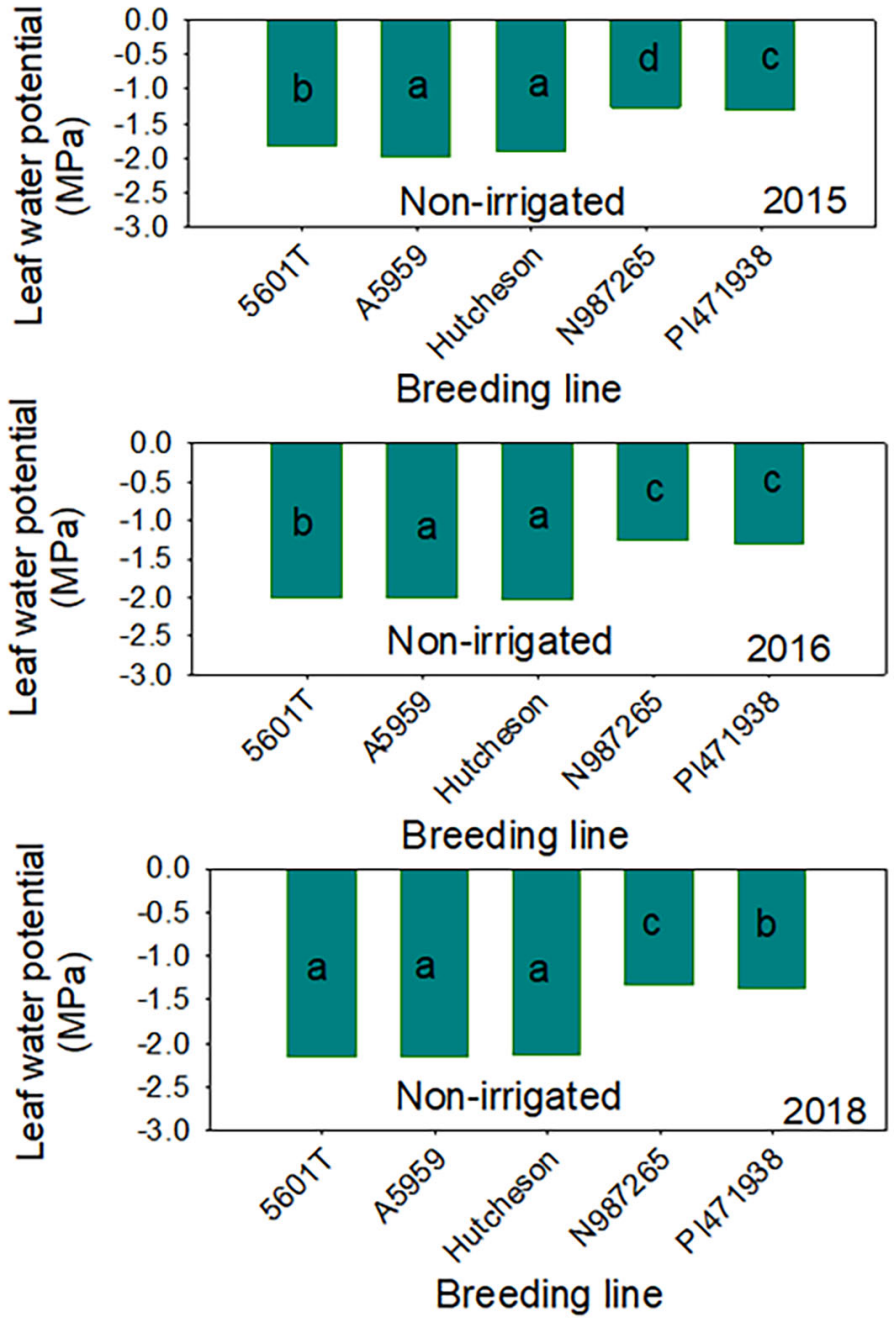
Leaf water potential (Ψw) under non-irrigated conditions in slow-wilting (SW) (N987265 and PI471938) and fast-wilting (FW) breeding lines/genotypes. Hutcheson, FW parent; PI471938, SW parent; N987265, SW progeny; FW, fast-wilting; SW, slow-wilting. Different letters indicate significant differences at P≤0.05; same letters indicate that there was no significant differences at P≤0.05.

The higher protein concentrations in SW genotypes under non-irrigation conditions could be a response to water stress or lower seed size (small seeds), as the yield of SW genotypes were consistently lower in each year. The higher concentrations of raffinose in 1 year out of 3 years and the higher concentrations of stachyose concentrations in SW genotypes under non-irrigation in all years consistently indicate the involvement of this sugar in water stress (drought tolerance indicator) for SW genotypes. Previous research in a repeated greenhouse experiment using NC-Roy (fast wilting), Boggs (intermediate wilting), and NTCPR94–5157 and N04-9646 (slow wilting, SW) genotypes showed that, under drought stress, nitrogen fixation and nitrogen assimilation were higher in NTCPR94–5157 and N04–9646 than in NC-Roy and Boggs. However, under severe water stress, nitrogen assimilation was low in all genotypes. This may indicate that SW genotypes had the ability to continue to perform both nitrogen fixation and assimilation at a higher rate compared with fast- or normal-wilting genotypes. In addition, it was shown that the concentrations of leaf and seed concentrations of K, P, Ca, Cu, Na, and B were higher in SW genotypes compared with fast or normal SW genotypes, suggesting the involvement of these mineral in water stress. It was suggested that K, P, Ca, Cu, Na, and B may be involved in SW trait to maintain homeostasis and osmotic regulation under water stress. However, to maintain leaf turgor pressure under water stress, the SW genotypes NTCPR94–5157 and N04–9646 kept a higher leaf water potential to conserve water as a mechanism strategy. Although the role of macro-nutrients (S, Ca, K, Mg, and P) or micronutrients (Fe, B, and Zn) in the physiological processes and biochemical pathways have been previously reported (Mengel and Kirkby, 1982; Marschner, 2012), the physiological and biochemical roles of these mineral nutrients in SW genotypes under drought stress have not been well investigated. Leaf water potential (**Figures 6**, **7**) showed that, under non-irrigated conditions, both SW genotypes had the ability to maintain a higher water potential (-1.20 Ψw) compared with those of FW where LWP dropped significantly to -2.77 Ψw, reflecting that the SW genotype could conserve water under drying soil (non-irrigated conditions). Our results are not in agreement with those of Bellaloui et al. (2013) who showed under greenhouse conditions that seed protein and sugars (sucrose and raffinose) were higher in SW genotypes. In addition, the current research showed that yield in SW genotypes was lower under irrigated and non-irrigated conditions in each year, and this is in disagreement with those reported by others (King et al., 2009; Ries et al., 2012; Sinclair et al., 2010). The conflicting results could be due to the differences in the growing conditions between our field experiments and those of others [King et al. (2009); Ries et al. (2012); Sinclair et al. (2010)] for heat and drought (**Figure 2**) and soil type as SW trait PIs were developed under lighter soil type compared to those of Mississippi delta of Sharkey, heavy, clay soils. The conflicting results could also be due to another possibility in that our SW lines have a low yield potential due to their exotic genetic background, as the exotic germplasms tend to yield less than commercial ones (Fletcher et al., 2007). Although our experiment was somewhat unique in that we actually replicated the irrigation treatment, that is, we did not just have replications within a drought block and an irrigation block (which does not replicate the irrigation treatment), but we actually replicated the irrigation treatment, having four total blocks with irrigation assigned to two blocks and non-irrigation assigned to two blocks (**Supplementary Figure S1**). Therefore, it is possible that the drought responses of SW lines under our field conditions for yield were different from those who found a higher yield under drought/non-irrigated conditions in SW lines. Therefore, further research is needed by conducting repeated field experiments using more SW lines across multiple environments and years under irrigated and non-irrigated conditions, and this needs enormous human and financial resources. Mechanisms involved in plant responses to drought stress are still not well understood (Hufstetler et al., 2007; Charlson et al., 2009; King et al., 2009)—for example, Sloane et al. (1990) reported that the wilting response (for FW or SW) of soybean genotypes to drought stress differ. It is clear, however, that SW genotypes have the ability to conserve soil moisture (Fletcher et al., 2007; King et al., 2009; Ries et al., 2012) by lowering the transpiration rate to maintain turgor pressure (Charlson et al., 2009) and guarantee the onset of the physiological process and biochemical pathways to assure growth, development, and yield during soil drying period. It was found, under field conditions, that the volumetric soil water content in SW genotypes was higher in SW genotypes compared with those of FW, but wilting responded similarly for both genotypes (King et al., 2009). On the other hand, under growth chamber conditions, transpiration declined similarly in response to drought stress for fast- and slow-wilting genotypes, reflecting that more than one mechanism is involved in SW trait (King et al., 2009). Using a SW genotype PI 416937 and FW cultivar Forrest, Sloane et al. (1990) found that water stress resulted in low leaf water potential, and it was equal for both SW and FW genotypes, but SW 416937 maintained lower levels of solute potential and a higher pressure potential than Forrest, indicating that PI 416937 can accumulate more solutes in leaves than Forrest under drought stress.

Therefore, a possible way to explain the conflicting results and reconcile the current findings with the previous ones can be in the following way: The slow wilting trait is due to the leaves which reduce their stomatal opening to reduce water loss and conserve water through evapotranspiration reduction. However, when they close or reduce their stomata, the rate of CO_2_ intake will be automatically reduced, decreasing the photosynthesis process and thus leading to less carbohydrate production and less biomass and yield. Therefore, we might expect the SW lines to be lower yielding than the lines that leave their stomata open. Another possible reason for the SW genotype to yield less than commercial checks is that the PI is exotic and its progeny is a line derived from it, and so it is likely that the exotic material brings a lot of “baggage/drag” genes that cause yield loss. It is common that cultivars have been selected for decades to not have the “baggage” genes that cause yield loss. Therefore, this means more cycles of crossing and selection to produce a “cultivar” that also has the SW trait. The third possibility is that the SW lines are environment specific in that their responses to drought are affected by environmental factors and growing conditions such as soil, temperature, and severity of drought; therefore, they did not respond to drought as previously reported by others. Although our findings show that higher seed oil in SW genotypes under irrigated conditions is beneficial for seed processing and higher protein, oleic acid, and stachyose in SW genotypes under non-irrigated conditions can be used as biomarkers for drought tolerance selection, the SW genotypes showed a consistent lower yield compared with the commercial checks. Therefore, while drought tolerance genotypes, tested under our conditions of Mississippi delta, expressed the drought trait and drought tolerance response mechanism (based on leaf water potential; conserve water), the yield was lower compared with commercial genotypes. Therefore, there is a trade-off between drought tolerance and yield in SW genotypes under some growing conditions or regions that may not be adapted to. In addition to this, the low yield potential is associated with the inherited genetic background “drag” associated with the exotic germplasm. If this is not the case, SW genotypes may not be able to over-yield commercial varieties. This genetic drag may be expressed more under the Mississippi delta growing conditions. Therefore, one possibility is to perform further selections to these SW genotypes to minimize the genetic background of low yield potential and increase their high yield potential. In spite of this, SW genotypes may still offer a strategic option to grow them under growing conditions adapted to their growing conditions that yield best.

When plants experienced drought, plant roots transmit signals to the shoot, triggering the accumulation of phytohormones such as abscisic acid (ABA) (Takahashi et al., 2020) to regulate stomatal closure to reduce water evaporation to reduce water loss. The activation of ABA signaling system must be inhibited for plant recovery (Kavi Kishor et al., 2022). It was suggested that the activated ABA signaling is terminated by the catabolism of ABA and the inactivation of ABA signaling mechanism that involve ABA degradation through hydrolysis and oxidative degradation (García-León et al., 2019). This process involves receptors and enhancers of ABA to inhibit ABA signaling (García-León et al., 2019). The rates of ABA biosynthesis, catabolism, and transport and the sensitivity of tissues to ABA together influence physiological changes in plants. Although stomatal closure is mainly induced by ABA signaling, other phytohormones such as jasmonic acid, salicylic acid, and ethylene were thought to involve in stomatal closure under drought by activating reactive oxygen species or nitric oxide accumulation (Agurla et al., 2018). Phytohormones such as strigolactone and cytokinin have been reported to regulate stomatal closure (Nguyen et al., 2016). As a result of stomatal closure and reduced stomatal conductance, CO_2_ intake is reduced, leading to reduced photosynthesis process (Zia et al., 2021) through the inhibition of its enzyme activity and reduced photochemical efficiency of photosystem II (Salehi-Lisar and Bakhshayeshan-Agdam, 2016). Under these conditions, the production of photosynthetic pigments such as chlorophylls and carotenoids involved in harvesting light energy will be reduced, leading to reduction in ATP and NADPH (important energy source and reducing power in the metabolism process). In addition to the physiological and signaling system changes during drought, gene expression occurs through up- and downregulation of genes involved in the drought response mechanism. As a consequence of all these changes at the physiological, biochemical, genetic, and molecular levels, loss of biomass and yield occur (Ye et al., 2019; Melandri et al., 2020; Yang et al., 2023). Drought-tolerant genotypes should possess a response mechanism to adjust against water deficit by delaying the drought response mechanism longer (slow wilting), reducing stomatal opening, stomatal conductance, evapotranspiration rate, and maintaining cell turgor to conserve water and trigger the transduction and signaling system, as explained above. This adaptation will allow for photosynthesis activity, production of metabolite, growth, and development and yields production. Drought-tolerant genotypes respond to drought using one or multiple mechanisms as cited above to adapt to water deficit and allow for slow normal growth, development, and yield production. It appears that for our SW genotypes, the drought response under our field experiment conditions was not favorable to the drought mechanism response to be expressed, leading to lack of adaptation and resulting in yield loss compared with FW genotypes. The lower yield in SW genotypes compared with FW genotypes is due to the fact that FW genotypes are checks and commercial varieties that were bred for high yield and do not possess genetic drags for low yield potential associated with exotic germplasm as in SW genotypes. Therefore, SW genotypes had lower yield than FW genotypes. These concepts were included in the discussion and conclusions.

**Figure 8:**
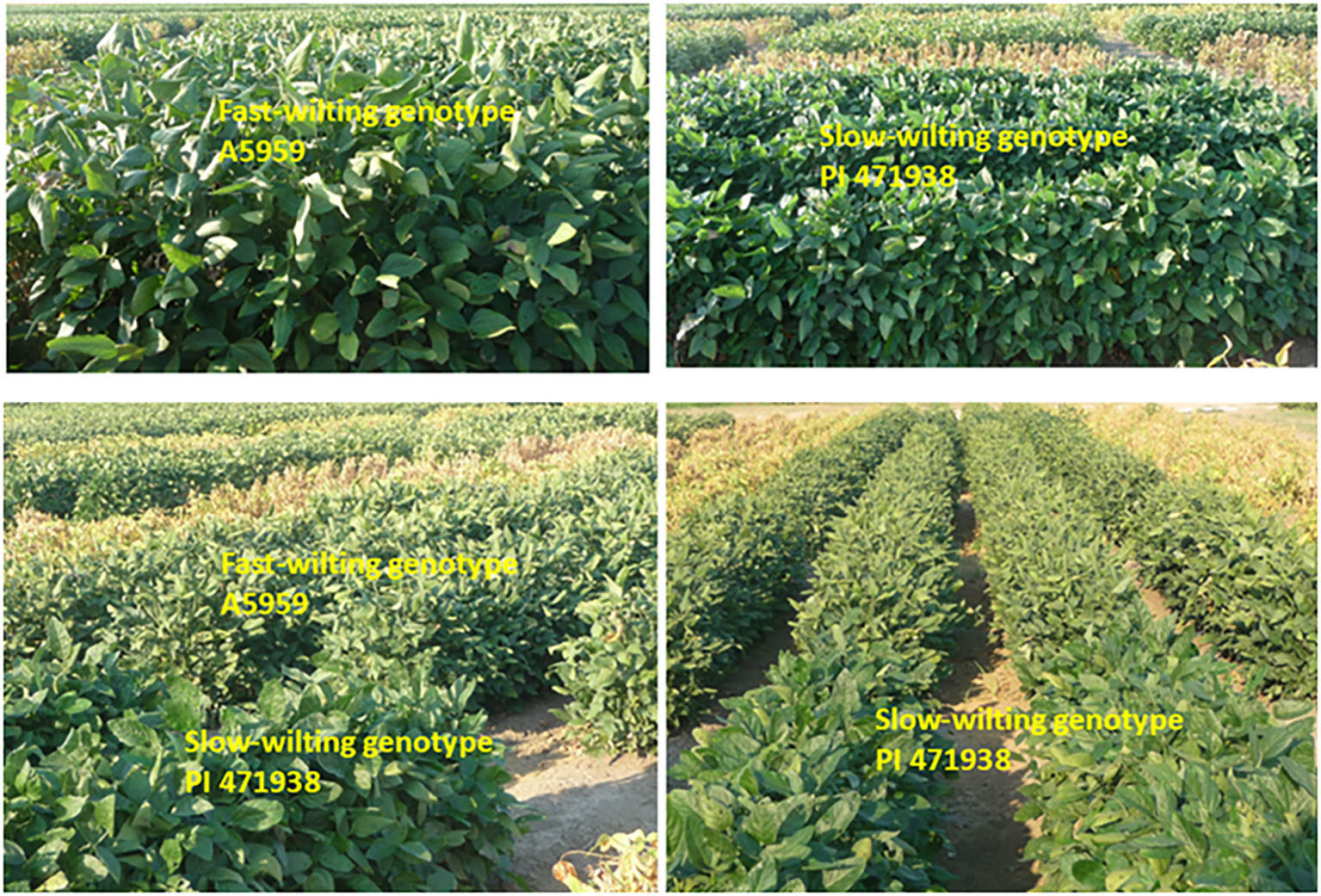
Fast-wilting genotypes (A5959) with leaves rolled on themselves under non-irrigated conditions compared with those of slow-wilting genotypes (PI471938).

**Figure 9:**
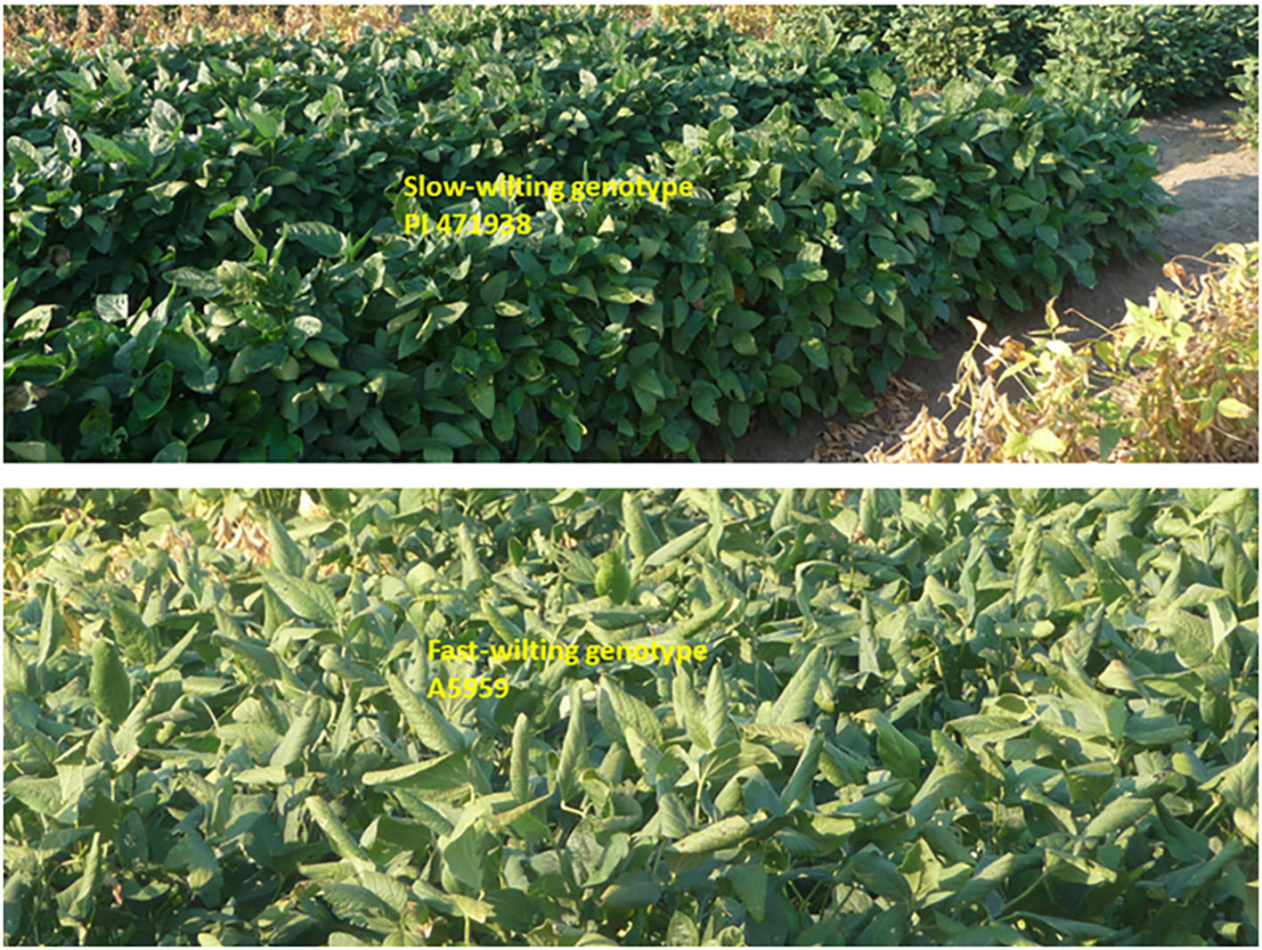
Fast-wilting genotypes (A5959) with leaves rolled on themselves (bottom) compared with slow-wilting genotypes (PI471938) (top). This response occurred under non-irrigated conditions.

The current research showed that leaf water potential in FW was lower and dropped to about -2.7 Ψw in FW genotypes compared to SW genotypes that showed ability to maintain higher leaf water potential (-1.10 Ψw) (**Figures 8**, **9**), indicating that WLP is one response mechanism for soybean to cope with drought stress in SW genotypes. However, LWP was not enough to explain the physiological or genetic response for lower yield in SW genotypes under irrigated and non-irrigated conditions in all years or over the 3 years (**Table 4**, **Figure 5**). Correlation between LWP and sugars and between LWP and yield showed inconsistent correlations, or no correlation was found between LWP and the sugars or between LWP and yield (**Supplementary Table S2**). Previous research on stress response mechanisms at the physiological, biochemical, and genetic levels showed that stomatal control has been considered as a major physiological indicator. It was shown that stomatal conductance in soybean was lower by 42% in drought-stressed leaves than normal leaves (Makbul et al., 2011). MG/BR46 (drought-tolerant soybean variety) showed fast decrease in stomatal conductance compared with BR16 (drought-susceptible variety). Prolonging drought stress showed no significant impact on the stomatal conductance of MG/BR16. Similar studies provided evidence that drought-tolerant soybean genotypes (C12 and W05) showed a higher reduction in stomatal conductance than the susceptible one (C08) (Hossain et al., 2014). Other physiological and biochemical indicators for drought tolerance are the maintenance of cell turgidity and water use efficiency (Hossain et al., 2015). Plant introduction PI 416937 (SW genotype) limits transpiration rate and maintains a lower osmotic potential to cope with drought stress. Furthermore, an increase in WUE was achieved by a drought-tolerant genotype (C12) by regulating stomatal closure during soil drying period (Hossain et al., 2014). The maintenance of cell turgidity under water-limited conditions was achieved by adjusting the osmotic potential due to the accumulation of proline, sucrose, soluble carbohydrates, glycine betaine, and other solutes (Hernández et al., 2001). As well known, under drought stress, plants produce ROS, for example, superoxide radical (O_2_−), hydroxyl radical (OH˙), and hydrogen peroxide (H_2_O_2_), and this is one of the biochemical responses causing damage to DNA, proteins, and lipids (Clement et al., 2008). The toxicity effects by ROS can be limited by antioxidant enzymes such as superoxide dismutase, catalase, and glutathione peroxidase and non-enzymatic scavengers (Creissen and Mullineaux, 2002; Aroca et al., 2003). It was found that drought stress increased the activities of catalase, glutathione reductase, and superoxide dismutase in soybean, limiting yield loss (Masoumi et al., 2010). Our current research showed that stachyose is a major bio-indicator for drought tolerance as this stachyose concentration was higher in SW genotypes in each year under non-irrigated conditions, indicating a possible use of stachyose as a drought tolerance biochemical compound indicator. This may be a response mechanism to drought stress. Previous research reported that, although the real biological function of raffinose and stachyose are not completely clear (Ren et al., 2009), both raffinose and stachyose are required for the acquisition of desiccation tolerance during seed development and maturation to protect seeds during seed dehydration and aging and probably for seed survival and storability (Obendorf, 1997). Although our findings indicate that higher seed oil in SW genotypes under irrigated conditions is beneficial for seed processing and higher protein, oleic acid, and stachyose in SW genotypes under non-irrigated conditions can be used as biomarkers for drought tolerance selection, the SW genotypes showed a consistent lower yield compared with the commercial checks. Therefore, while drought tolerance genotypes, tested under our conditions of Mississippi delta, expressed the drought trait and drought tolerance response mechanism (based on leaf water potential; conserve water), the yield was lower compared with commercial genotypes. Therefore, there is a trade-off between drought tolerance and yield in SW genotypes under some growing conditions or regions that they may not be adapted to. In addition to this, the low yield potential is associated with the inherited genetic background “drag” associated with the exotic germplasm. If this is not the case, SW genotypes may not be able to over-yield commercial varieties. This genetic drag may be expressed more under the Mississippi delta growing conditions. Therefore, one possibility is to perform further selections to these SW genotypes to minimize the genetic background of low yield potential and increase their high yield potential. In spite of this, SW genotypes may still offer a strategic option to grow them under growing conditions adapted to their growing conditions that yield best.

## 4 Limitation of the current study findings and how the findings contribute to the breeding program to improve drought tolerance

Our findings will help the scientific communities further understand the physiological and genetic response mechanisms of drought tolerance in soybean for yield and seed composition/nutrition. Our findings showing that higher seed oil in SW genotypes under irrigated conditions (beneficial for seed processing) and higher protein, oleic acid, and stachyose in SW genotypes under non-irrigated conditions can be a useful tool for breeders to use as biomarkers for drought tolerance selection. The findings that SW genotypes showed a consistent decrease in yield compared with the commercial checks are an important observation and were not unexpected. The consistent decreases in yield in SW genotypes compared with the FW genotypes indicated that the expression of SW trait and its response to drought for yield differs, and it depends on genotype, maturity group, and environmental growing conditions, especially temperature, humidity, drought severity, and their interactions. Furthermore, this provides breeding programs with new findings on the relationship between drought and low yield potential of some SW genotypes due to the genetic drag derived from the drought-tolerant exotic germplasm. These findings are integrated into the breeders’ selection criteria by using advanced technologies in genomics, transcriptomics, and genetic engineering. Genomics and transcriptomics will allow for the identification of genes and genomic regions (QTL and molecular markers) associated with drought and yield potential using either isoline sets for drought tolerance rather than using exotic PIs or progenies that possess low yield drag. Future research will focus on conducting field experiments with a reasonable size of isolines across locations and across years to test the trait stability and minimize the interaction of the environmental factors with drought genes using advanced technologies in genomics and transcriptomics to identify genes, genomic regions, and molecular markers associated with drought and high-yield-potential genotypes. These markers can be used for the breeding selection. On the other hand, exotic germplasm that genetically possesses low yield potential could be further selected to replace the low potential genetic drag and produce a drought-tolerant variety with high yield potential. The findings of higher seed protein, oleic acid, and stachyose sugar in SW genotypes than checks under non-irrigated conditions are important and will provide breeders a soybean selection with higher seed nutritional qualities under drought conditions.

## 5 Conclusions

The current research demonstrated that protein in SW genotypes was lower, and oil was higher than those of checks under irrigated conditions. This pattern changed, especially for protein and oil when soybean had been grown under non-irrigated conditions in that protein, oleic acid, and stachyose sugar were higher in SW genotypes than in checks. The increase of protein in non-irrigation could be due to seed size and lower seed yield of SW genotypes under non-irrigated conditions. Seed yield was lower in SW compared with those of FW genotypes under irrigated and non-irrigated conditions, indicating that the drought trait lines may still carry its genetic drag for low yield potential, and further selections are needed to minimize its influence on yield potential. Moreover, it may indicate that the drought trait and its gene expression could be environment specific. Therefore, the response mechanism of SW trait could be due to multiple response mechanisms as leaf water potential in the current research could explain the alteration of seed protein, oil, oleic acid, and sugars but could not explain the significant lower yield in SW genotypes under irrigated and non-irrigated conditions in all years. This requires further research using a bigger size of lines across years and across locations which is also important. Furthermore, using isolines for drought tolerance to avoid the confounding effect between genotype and SW trait or using appropriate RIL populations to further understand the physiological and genetic factors controlling SW trait is also important. The higher stachyose in SW genotypes under non-irrigated conditions may indicate the involvement of the sugar stachyose in drought tolerance in protecting the seed against dehydration and can be used as a biochemical marker for drought tolerance, and they can be used as biomarkers for breeding selections for drought tolerance.

## Data Availability

The original contributions presented in the study are included in the article/**Supplementary Material**. Further inquiries can be directed to the corresponding author.
