# Supplementary material for: Seed yield and nutrition in slow-wilting soybean breeding lines as influenced by irrigated and non-irrigated conditions in the Midsouth USA
Source: Front Plant Sci. 2026 Jan 6;16:1662965. doi: 10.3389/fpls.2025.1662965 (PMC12816249; doi:10.3389/fpls.2025.1662965)
Supplement: Supplementary file 1 [file Image1.pdf]

Supplementary materials

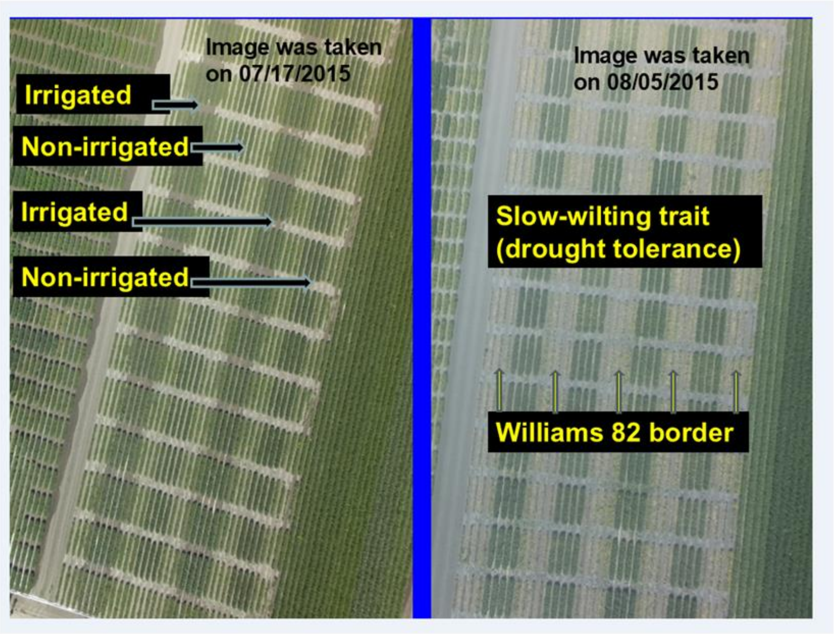

Figure S1. Shows the irrigated and non-irrigated plots in a repeated irrigation and non-irrigated field experiment. The image was taken in 2015.
