# Supplementary material for: Seed yield and nutrition in slow-wilting soybean breeding lines as influenced by irrigated and non-irrigated conditions in the Midsouth USA
Source: Front Plant Sci. 2026 Jan 6;16:1662965. doi: 10.3389/fpls.2025.1662965 (PMC12816249; doi:10.3389/fpls.2025.1662965)
Supplement: Supplementary file 2 [file Table1.docx]

Table S1. Irrigation water in 2015, 2016, and 2018. The table shows that 2016 was the drier (six irrigations during the growing season), and 2018 was the wetter (only three times was used).

|  |  | **Year 2015** |  |  |  |  |
| --- | --- | --- | --- | --- | --- | --- |
|  |  | **Irrigation Usage Data** |  |  |  |  |
|  |  |  |  |  |  |  |
| **Field** | **Date** | **Approx. Hours** | **Beginning Reading** | **Ending Reading** | **Gallons Used** | **RPM** |
| 2 North | 6/11-12/2015 | 19.5 hrs | 33,215,400 | 34,121,700 | 906,300 | 1600 |
| 2 North | 7/15-16/2015 | 23 hrs | 34,121,700 | 35,184,500 | 1,062,800 | 1600 |
| 2 North | 7/27-28/2015 | 23 hrs | 35,184,500 | 36,234,400 | 1,049,900 | 1600 |
| 2 North | 8/6-7/2015 | 23 hrs. | 36,234,400 | 37,265,900 | 1,031,500 | 1600 |
| 2 North | 8/26-27/2015 | 24 hrs | 37,265,900 | 38,391,500 | 1,125,600 | 1600 |
| 2 North | 9/17-18/2015 | 24 hrs | 38,391,500 | 39,517,200 | 1,125,700 | 1600 |
|  |  |  |  |  |  |  |
|  |  |  |  | **TOTAL=** | **6,301,800** |  |
|  |  |  |  |  |  |  |
|  |  |  |  |  |  |  |
|  |  |  |  |  |  |  |
| **Field** | **Date** | **Approx. Hours** | **Beginning Reading** | **Ending Reading** | **Gallons Used** | **RPM** |
| 17 | 6/12-13/2015 | 20 hrs | 33,108,100 | 34,194,300 | 1,086,200 | 1500 |
| 17 | 7/16-17/2015 | 19.5 hrs | 34,194,300 | 35,321,900 | 1,127,600 | 1500 |
| 17 | 7/28-29/2015 | 19 hrs | 35,321,900 | 36,383,200 | 1,061,300 | 1500 |
| 17 | 8/7-8/2015 | 19 hrs | 36,383,200 | 37,456,700 | 1,073,500 | 1500 |
| 17 | 8/28-29/2015 | 18 hrs | 37,456,700 | 38,377,300 | 920,600 | 1500 |
| 17 |  |  |  |  |  |  |
|  |  |  |  |  |  |  |
|  |  |  |  | **TOTAL=** | **5,269,200** |  |
|  |  | **Year 2016** |  |  |  |  |
|  |  | **Irrigation Usage Data** |  |  |  |  |
|  |  |  |  |  |  |  |
| **Field** | **Date** | **Approx. Hours** | **Beginning Reading** | **Ending Reading** | **Gallons Used** | **RPM** |
| 2 Center | 6/26-27/2016 | 25 hrs | 39,517,200 | 40,509,400 | 992,200 | 1500 |
| 2 Center | 7/18-19/2016 | 26 hrs. | 40,509,400 | 41,599,800 | 1,090,400 | 1500 |
| 2 Center | 9/16-17/2016 | 18hrs. | 41,599,800 | 42,231,500 | 631,700 | 1200 |
| 2 Center |  |  |  |  |  |  |
| 2 Center |  |  |  |  |  |  |
| 2 Center |  |  |  |  |  |  |
|  |  |  |  |  |  |  |
|  |  |  |  | **TOTAL=** | **2,714,300** |  |
|  |  |  |  |  |  |  |
|  |  |  |  |  |  |  |
|  |  |  |  |  |  |  |
| **Field** | **Date** | **Approx. Hours** | **Beginning Reading** | **Ending Reading** | **Gallons Used** | **RPM** |
| 17 | 5/27/2016 | 8 hrs | 38,377,300 | 38,842,900 | 465,600 | 1300 |
| 17 | 6/29-30/2016 | 24 hrs | 38,842,900 | 40,186,200 | 1,343,300 | 1500 |
| 17 | 7/22-23/2016 | 23 hrs | 40,186,200 | 41,408,900 | 1,222,700 | 1500 |
| 17 |  |  |  |  |  |  |
| 17 |  |  |  |  |  |  |
| 17 |  |  |  |  |  |  |
|  |  |  |  |  |  |  |
|  |  |  |  | **TOTAL=** | **3,031,600** |  |
|  |  | **Year 2018** |  |  |  |  |
|  |  | **Irrigation Usage Data** |  |  |  |  |
|  |  |  |  |  |  |  |
| **Field** | **Date** | **Approx. Hours** | **Beginning Reading** | **Ending Reading** | **Gallons Used** | **RPM** |
| 2 Center | 6/8-9/2018 | 26 hr | 44,967,600 | 46,049,300 | 1,081,700 | 1500 |
| 2 Center | 7/18-19/2018 | 27 hr | 46,049,300 | 47,204,700 | 1,155,400 | 1500 |
| 2 Center | 8/5-6/2018 | 23 | 47,204,700 | 48,131,400 | 926,700 | 1500 |
| 2 Center |  |  |  |  |  |  |
| 2 Center |  |  |  |  |  |  |
| 2 Center |  |  |  |  |  |  |
|  |  |  |  |  |  |  |
|  |  |  |  | **TOTAL=** | **3,163,800** |  |
|  |  |  |  |  |  |  |
|  |  |  |  |  |  |  |
|  |  |  |  |  |  |  |
| **Field** | **Date** | **Approx. Hours** | **Beginning Reading** | **Ending Reading** | **Gallons Used** | **RPM** |
| 17 | 6/8-9/2018 | 25 hr | 43,964,000 | 45,396,800 | 1,432,800 | 1500 |
| 17 | 8/6-7/2018 | 27 hr | 45,396,800 | 46,940,800 | 1,574,000 | 1500 |
| 17 |  |  |  |  |  |  |
| 17 |  |  |  |  |  |  |
| 17 |  |  |  |  |  |  |
| 17 |  |  |  |  |  |  |
|  |  |  |  |  |  |  |
|  |  |  |  | **TOTAL=** | **3,006,800** |  |
