# Supplementary material for: Seed yield and nutrition in slow-wilting soybean breeding lines as influenced by irrigated and non-irrigated conditions in the Midsouth USA
Source: Front Plant Sci. 2026 Jan 6;16:1662965. doi: 10.3389/fpls.2025.1662965 (PMC12816249; doi:10.3389/fpls.2025.1662965)
Supplement: Supplementary file 3 [file Table2.docx]

Table S2. Shows the correlation between leaf water potential (LWP) and sugars (mg/g) (sucrose, raffinose, and stachyose) in soybean fast-wilting (FW) checks (5601T, A5959, and Hutcheson); and slow-wiling (SW) breeding lines (N987265 and PI471938). The experiment was conducted in 2015, 2016, and 2018 in Stoneville, MS, USA in 2015, 2016, and 2018 under irrigated (irr) and non-irrigated (nonirr). In each cell of the table, the top value is R= correlation coefficient, and the bottom value is P=probability. Level of significance was P=≤0.05.

| **2015 irr 5601T** | |  |  |  |  |  | **2015 irr A5959** | |  |  |  |
| --- | --- | --- | --- | --- | --- | --- | --- | --- | --- | --- | --- |
|  |  |  |  |  |  |  |  |  |  |  |  |
|  | **sucrose** | **raffinose** | **stachyose** | **yield** |  |  |  | **sucrose** | **raffinose** | **stachyose** | **yield** |
| **raffinose** | R=0.65832 |  |  |  |  |  | **raffinose** | -0.77105 |  |  |  |
|  | P=0.1552 |  |  |  |  |  |  | 0.0726 |  |  |  |
| **stachyose** | 0.90088 | 0.60101 |  |  |  |  | **stachyose** | 0.2244 | -0.13879 |  |  |
|  | 0.0143 | 0.207 |  |  |  |  |  | 0.6691 | 0.7932 |  |  |
| **yield** | 0.89676 | 0.65078 | 0.74555 |  |  |  | **yield** | 0.7669 | -0.68382 | 0.23129 |  |
|  | 0.0154 | 0.1616 | 0.0889 |  |  |  |  | 0.0752 | 0.1342 | 0.6592 |  |
| **LWP** | 0.36772 | 0.78541 | 0.17292 | 0.61891 |  |  | **LWP** | -0.75024 | 0.79057 | -0.02601 | -0.61941 |
|  | 0.4733 | 0.0641 | 0.7432 | 0.1902 |  |  |  | 0.0858 | 0.0612 | 0.961 | 0.1897 |
|  |  |  |  |  |  |  |  |  |  |  |  |
|  |  |  |  |  |  |  |  |  |  |  |  |
| **2015 irr Hutcheson** | |  |  |  |  |  | **2015 irr N987265** | | |  |  |
|  |  |  |  |  |  |  |  |  |  |  |  |
|  | **sucrose** | **raffinose** | **stachyose** | **yield** |  |  |  | **sucrose** | **raffinose** | **stachyose** | **yield** |
| **raffinose** | -0.85677 |  |  |  |  |  | **raffinose** | 0.39437 |  |  |  |
|  | 0.0293 |  |  |  |  |  |  | 0.4391 |  |  |  |
| **stachyose** | 0.42541 | -0.2497 |  |  |  |  | **stachyose** | 0.03437 | 0.0808 |  |  |
|  | 0.4004 | 0.6332 |  |  |  |  |  | 0.9485 | 0.8791 |  |  |
| **yield** | 0.70611 | -0.63704 | 0.05348 |  |  |  | **yield** | 0.08024 | 0.79054 | -0.34728 |  |
|  | 0.1169 | 0.1737 | 0.9199 |  |  |  |  | 0.8799 | 0.0612 | 0.5 |  |
| **LWP** | 0.88645 | -0.70619 | 0.40819 | 0.65063 |  |  | **LWP** | -0.79245 | -0.08048 | -0.01989 | 0.02277 |
|  | 0.0186 | 0.1168 | 0.4217 | 0.1618 |  |  |  | 0.0601 | 0.8795 | 0.9702 | 0.9659 |
|  |  |  |  |  |  |  |  |  |  |  |  |
|  |  |  |  |  |  |  |  |  |  |  |  |
| **2015 irr PI471938** | |  |  |  |  |  |  |  |  |  |  |
|  |  |  |  |  |  |  |  |  |  |  |  |
|  | **sucrose** | **raffinose** | **stachyose** | **yield** |  |  |  |  |  |  |  |
| **raffinose** | 0.90887 |  |  |  |  |  |  |  |  |  |  |
|  | 0.0121 |  |  |  |  |  |  |  |  |  |  |
| **stachyose** | 0.95562 | 0.97002 |  |  |  |  |  |  |  |  |  |
|  | 0.0029 | 0.0013 |  |  |  |  |  |  |  |  |  |
| **yield** | 0.33678 | 0.49089 | 0.53231 |  |  |  |  |  |  |  |  |
|  | 0.5139 | 0.3228 | 0.277 |  |  |  |  |  |  |  |  |
| **LWP** | 0.5822 | 0.24744 | 0.4647 | 0.22372 |  |  |  |  |  |  |  |
|  | 0.2254 | 0.6364 | 0.3531 | 0.67 |  |  |  |  |  |  |  |
|  |  |  |  |  |  |  |  |  |  |  |  |
|  |  |  |  |  |  |  |  |  |  |  |  |
| **2015 nonirr 5601T** | |  |  |  |  |  | **2015 nonirr A5959** | | |  |  |
|  |  |  |  |  |  |  |  |  |  |  |  |
|  | **sucrose** | **raffinose** | **stachyose** | **yield** |  |  |  | **sucrose** | **raffinose** | **stachyose** | **yield** |
| **raffinose** | -0.16433 |  |  |  |  |  | **raffinose** | 0.31981 |  |  |  |
|  | 0.7557 |  |  |  |  |  |  | 0.5366 |  |  |  |
| **stachyose** | 0.87737 | 0.0429 |  |  |  |  | **stachyose** | 0.70157 | 0.39231 |  |  |
|  | 0.0216 | 0.9357 |  |  |  |  |  | 0.1203 | 0.4417 |  |  |
| **yield** | -0.33261 | -0.07747 | -0.0394 |  |  |  | **yield** | -0.13369 | -0.18728 | 0.16355 |  |
|  | 0.5195 | 0.884 | 0.9409 |  |  |  |  | 0.8007 | 0.7224 | 0.7569 |  |
| **LWP** | 0.11149 | 0.09366 | 0.48954 | 0.43078 |  |  | **LWP** | -0.50814 | -0.02354 | -0.19537 | -0.58277 |
|  | 0.8335 | 0.8599 | 0.3244 | 0.3938 |  |  |  | 0.3034 | 0.9647 | 0.7107 | 0.2248 |
|  |  |  |  |  |  |  |  |  |  |  |  |
|  |  |  |  |  |  |  |  |  |  |  |  |
| **2015 nonirr Hutcheson** | | |  |  |  |  | **2015 nonirr N987265** | | |  |  |
|  |  |  |  |  |  |  |  |  |  |  |  |
|  | **sucrose** | **raffinose** | **stachyose** | **yield** |  |  |  | **sucrose** | **raffinose** | **stachyose** | **yield** |
| **raffinose** | -0.27226 |  |  |  |  |  | **raffinose** | -0.26688 |  |  |  |
|  | 0.6017 |  |  |  |  |  |  | 0.6092 |  |  |  |
| **stachyose** | 0.91222 | -0.4645 |  |  |  |  | **stachyose** | -0.28806 | 0.64398 |  |  |
|  | 0.0112 | 0.3534 |  |  |  |  |  | 0.5799 | 0.1676 |  |  |
| **yield** | -0.13597 | -0.35951 | -0.01787 |  |  |  | **yield** | 0.13367 | 0.30326 | 0.16067 |  |
|  | 0.7973 | 0.484 | 0.9732 |  |  |  |  | 0.8007 | 0.5591 | 0.7611 |  |
| **LWP** | 0.76771 | 0.25849 | 0.58786 | -0.27039 |  |  | **LWP** | -0.27657 | 0.40738 | 0.73398 | -0.06118 |
|  | 0.0747 | 0.6209 | 0.2198 | 0.6043 |  |  |  | 0.5957 | 0.4227 | 0.0967 | 0.9083 |
|  |  |  |  |  |  |  |  |  |  |  |  |
|  |  |  |  |  |  |  |  |  |  |  |  |
| **2015 nonirr PI471938** | | |  |  |  |  |  |  |  |  |  |
|  |  |  |  |  |  |  |  |  |  |  |  |
|  | **sucrose** | **raffinose** | **stachyose** | **yield** |  |  |  |  |  |  |  |
| **raffinose** | 0.3258 |  |  |  |  |  |  |  |  |  |  |
|  | 0.5286 |  |  |  |  |  |  |  |  |  |  |
| **stachyose** | 0.95639 | 0.16617 |  |  |  |  |  |  |  |  |  |
|  | 0.0028 | 0.753 |  |  |  |  |  |  |  |  |  |
| **yield** | 0.06487 | -0.20651 | 0.15095 |  |  |  |  |  |  |  |  |
|  | 0.9028 | 0.6946 | 0.7753 |  |  |  |  |  |  |  |  |
| **LWP** | -0.17856 | 0.38478 | -0.16682 | 0.70245 |  |  |  |  |  |  |  |
|  | 0.735 | 0.4513 | 0.7521 | 0.1196 |  |  |  |  |  |  |  |
|  |  |  |  |  |  |  |  |  |  |  |  |
|  |  |  |  |  |  |  |  |  |  |  |  |
| **2016 irr 5601T** | |  |  |  |  |  | **2016 irr A5959** | |  |  |  |
|  |  |  |  |  |  |  |  |  |  |  |  |
|  | **sucrose** | **raffinose** | **stachyose** | **yield** |  |  |  | **sucrose** | **raffinose** | **stachyose** | **yield** |
| **raffinose** | -0.06711 |  |  |  |  |  | **raffinose** | -0.55196 |  |  |  |
|  | 0.8995 |  |  |  |  |  |  | 0.2561 |  |  |  |
| **stachyose** | -0.13996 | -0.43786 |  |  |  |  | **stachyose** | 0.56949 | -0.39675 |  |  |
|  | 0.7914 | 0.3852 |  |  |  |  |  | 0.2381 | 0.4361 |  |  |
| **yield** | -0.4444 | 0.19822 | -0.66085 |  |  |  | **yield** | -0.75979 | 0.3287 | -0.19043 |  |
|  | 0.3773 | 0.7066 | 0.153 |  |  |  |  | 0.0796 | 0.5247 | 0.7178 |  |
| **LWP** | -0.73527 | -0.51235 | 0.48031 | 0.14405 |  |  | **LWP** | 0.45916 | -0.10825 | 0.34593 | 0.10434 |
|  | 0.0958 | 0.2987 | 0.3349 | 0.7854 |  |  |  | 0.3597 | 0.8383 | 0.5018 | 0.8441 |
|  |  |  |  |  |  |  |  |  |  |  |  |
|  |  |  |  |  |  |  |  |  |  |  |  |
| **2016 irr Hutcheson** | |  |  |  |  |  | **2016 irr N987265** | | |  |  |
|  |  |  |  |  |  |  |  |  |  |  |  |
|  | **sucrose** | **raffinose** | **stachyose** | **yield** |  |  |  | **sucrose** | **raffinose** | **stachyose** | **yield** |
| **raffinose** | 0.12712 |  |  |  |  |  | **raffinose** | -0.44496 |  |  |  |
|  | 0.8103 |  |  |  |  |  |  | 0.3766 |  |  |  |
| **stachyose** | 0.30311 | 0.02499 |  |  |  |  | **stachyose** | 0.13441 | -0.36445 |  |  |
|  | 0.5593 | 0.9625 |  |  |  |  |  | 0.7996 | 0.4775 |  |  |
| **yield** | -0.16456 | 0.88468 | -0.32244 |  |  |  | **yield** | 0.75974 | -0.05308 | 0.51353 |  |
|  | 0.7554 | 0.0192 | 0.5331 |  |  |  |  | 0.0797 | 0.9205 | 0.2974 |  |
| **LWP** | -0.59348 | -0.57607 | 0.07591 | -0.56878 |  |  | **LWP** | 0.19706 | 0.59976 | 0.22827 | 0.68109 |
|  | 0.2143 | 0.2315 | 0.8864 | 0.2388 |  |  |  | 0.7082 | 0.2082 | 0.6635 | 0.1363 |
|  |  |  |  |  |  |  |  |  |  |  |  |
|  |  |  |  |  |  |  |  |  |  |  |  |
| **2016 irr PI471938** | |  |  |  |  |  |  |  |  |  |  |
|  |  |  |  |  |  |  |  |  |  |  |  |
|  | **sucrose** | **raffinose** | **stachyose** | **yield** |  |  |  |  |  |  |  |
| **raffinose** | 0.23989 |  |  |  |  |  |  |  |  |  |  |
|  | 0.6471 |  |  |  |  |  |  |  |  |  |  |
| **stachyose** | 0.64696 | 0.12419 |  |  |  |  |  |  |  |  |  |
|  | 0.165 | 0.8147 |  |  |  |  |  |  |  |  |  |
| **yield** | -0.49494 | -0.26624 | 0.25931 |  |  |  |  |  |  |  |  |
|  | 0.3182 | 0.6101 | 0.6197 |  |  |  |  |  |  |  |  |
| **LWP** | -0.34981 | -0.31289 | 0.00694 | 0.75234 |  |  |  |  |  |  |  |
|  | 0.4967 | 0.546 | 0.9896 | 0.0844 |  |  |  |  |  |  |  |
|  |  |  |  |  |  |  |  |  |  |  |  |
|  |  |  |  |  |  |  |  |  |  |  |  |
| **2016 nonirr 5601T** | |  |  |  |  |  | **2016 nonirr A5959** | | |  |  |
|  |  |  |  |  |  |  |  |  |  |  |  |
|  | **sucrose** | **raffinose** | **stachyose** | **yield** |  |  |  | **sucrose** | **raffinose** | **stachyose** | **yield** |
| **raffinose** | -0.32349 |  |  |  |  |  | **raffinose** | -0.31514 |  |  |  |
|  | 0.5317 |  |  |  |  |  |  | 0.5429 |  |  |  |
| **stachyose** | -0.48003 | 0.11575 |  |  |  |  | **stachyose** | -0.69674 | 0.75117 |  |  |
|  | 0.3353 | 0.8271 |  |  |  |  |  | 0.124 | 0.0852 |  |  |
| **yield** | -0.29541 | 0.58037 | -0.36802 |  |  |  | **yield** | 0.57415 | -0.00508 | -0.43877 |  |
|  | 0.5698 | 0.2272 | 0.4729 |  |  |  |  | 0.2334 | 0.9924 | 0.3841 |  |
| **LWP** | -0.0184 | 0.67254 | -0.37247 | 0.26442 |  |  | **LWP** | 0.13926 | 0.30548 | -0.08701 | 0.76262 |
|  | 0.9724 | 0.1433 | 0.4671 | 0.6126 |  |  |  | 0.7925 | 0.556 | 0.8698 | 0.0778 |
|  |  |  |  |  |  |  |  |  |  |  |  |
|  |  |  |  |  |  |  |  |  |  |  |  |
| **2016 nonirr Hutcheson** | | |  |  |  |  | **2016 nonirr N987265** | | |  |  |
|  |  |  |  |  |  |  |  |  |  |  |  |
|  | **sucrose** | **raffinose** | **stachyose** | **yield** |  |  |  | **sucrose** | **raffinose** | **stachyose** | **yield** |
| **raffinose** | 0 |  |  |  |  |  | **raffinose** | 0.43749 |  |  |  |
|  | 1 |  |  |  |  |  |  | 0.3856 |  |  |  |
| **stachyose** | -0.67612 | 0.15005 |  |  |  |  | **stachyose** | -0.11916 | -0.12063 |  |  |
|  | 0.1404 | 0.7766 |  |  |  |  |  | 0.8221 | 0.8199 |  |  |
| **yield** | -0.13603 | 0.04351 | -0.35542 |  |  |  | **yield** | 0.69167 | 0.89519 | -0.42135 |  |
|  | 0.7972 | 0.9348 | 0.4893 |  |  |  |  | 0.1279 | 0.0159 | 0.4054 |  |
| **LWP** | 0.327 | 0.22942 | 0.12048 | 0.17301 |  |  | **LWP** | -0.30859 | -0.51704 | -0.2408 | -0.37848 |
|  | 0.527 | 0.6619 | 0.8202 | 0.7431 |  |  |  | 0.5518 | 0.2936 | 0.6458 | 0.4594 |
|  |  |  |  |  |  |  |  |  |  |  |  |
|  |  |  |  |  |  |  |  |  |  |  |  |
| **2016 nonirr PI471938** | | |  |  |  |  |  |  |  |  |  |
|  |  |  |  |  |  |  |  |  |  |  |  |
|  | **sucrose** | **raffinose** | **stachyose** | **yield** |  |  |  |  |  |  |  |
| **raffinose** | -0.1812 |  |  |  |  |  |  |  |  |  |  |
|  | 0.7312 |  |  |  |  |  |  |  |  |  |  |
| **stachyose** | 0.88437 | -0.1966 |  |  |  |  |  |  |  |  |  |
|  | 0.0193 | 0.7089 |  |  |  |  |  |  |  |  |  |
| **yield** | -0.48576 | 0.42881 | -0.46714 |  |  |  |  |  |  |  |  |
|  | 0.3287 | 0.3962 | 0.3503 |  |  |  |  |  |  |  |  |
| **LWP** | 0.46995 | -0.3644 | 0.6729 | -0.74911 |  |  |  |  |  |  |  |
|  | 0.347 | 0.4776 | 0.143 | 0.0865 |  |  |  |  |  |  |  |
|  |  |  |  |  |  |  |  |  |  |  |  |
|  |  |  |  |  |  |  |  |  |  |  |  |
| **2018 irr 5601T** | |  |  |  |  |  | **2018 irr A5959** | |  |  |  |
|  |  |  |  |  |  |  |  |  |  |  |  |
|  | **sucrose** | **raffinose** | **stachyose** | **yield** |  |  |  | **sucrose** | **raffinose** | **stachyose** | **yield** |
| **raffinose** | 0.71824 |  |  |  |  |  | **raffinose** | 0.17454 |  |  |  |
|  | 0.1079 |  |  |  |  |  |  | 0.7409 |  |  |  |
| **stachyose** | 0.20846 | -0.05114 |  |  |  |  | **stachyose** | 0.86865 | 0.03203 |  |  |
|  | 0.6918 | 0.9234 |  |  |  |  |  | 0.0247 | 0.952 |  |  |
| **yield** | 0.44848 | 0.32211 | -0.50973 |  |  |  | **yield** | 0.04081 | -0.2597 | 0.3865 |  |
|  | 0.3724 | 0.5336 | 0.3016 |  |  |  |  | 0.9388 | 0.6192 | 0.4491 |  |
| **LWP** | -0.16193 | -0.24914 | 0.84513 | -0.39769 |  |  | **LWP** | 0.1967 | -0.39131 | 0.0394 | -0.18905 |
|  | 0.7592 | 0.634 | 0.0341 | 0.4349 |  |  |  | 0.7088 | 0.443 | 0.9409 | 0.7198 |
|  |  |  |  |  |  |  |  |  |  |  |  |
|  |  |  |  |  |  |  |  |  |  |  |  |
| **2018 irr Hutcheson** | |  |  |  |  |  | **2018 irr N987265** | | |  |  |
|  |  |  |  |  |  |  |  |  |  |  |  |
|  | **sucrose** | **raffinose** | **stachyose** | **yield** |  |  |  | **sucrose** | **raffinose** | **stachyose** | **yield** |
| **raffinose** | 0.37463 |  |  |  |  |  | **raffinose** | -0.38604 |  |  |  |
|  | 0.4643 |  |  |  |  |  |  | 0.4497 |  |  |  |
| **stachyose** | 0.00086 | -0.54473 |  |  |  |  | **stachyose** | 0.72041 | -0.44129 |  |  |
|  | 0.9987 | 0.2637 |  |  |  |  |  | 0.1063 | 0.381 |  |  |
| **yield** | 0.07724 | 0.15176 | -0.49045 |  |  |  | **yield** | -0.32115 | -0.38811 | 0.23958 |  |
|  | 0.8844 | 0.7741 | 0.3233 |  |  |  |  | 0.5348 | 0.4471 | 0.6475 |  |
| **LWP** | -0.26728 | -0.3741 | -0.28824 | 0.44181 |  |  | **LWP** | 0.20743 | -0.46982 | 0.34805 | 0.43041 |
|  | 0.6086 | 0.465 | 0.5796 | 0.3804 |  |  |  | 0.6933 | 0.3471 | 0.499 | 0.3942 |
|  |  |  |  |  |  |  |  |  |  |  |  |
|  |  |  |  |  |  |  |  |  |  |  |  |
| **2018 irr PI471938** | |  |  |  |  |  |  |  |  |  |  |
|  |  |  |  |  |  |  |  |  |  |  |  |
|  | **sucrose** | **raffinose** | **stachyose** | **yield** |  |  |  |  |  |  |  |
| **raffinose** | -0.18328 |  |  |  |  |  |  |  |  |  |  |
|  | 0.7282 |  |  |  |  |  |  |  |  |  |  |
| **stachyose** | 0.53962 | 0.22361 |  |  |  |  |  |  |  |  |  |
|  | 0.2691 | 0.6702 |  |  |  |  |  |  |  |  |  |
| **yield** | -0.33155 | 0.38346 | 0.4342 |  |  |  |  |  |  |  |  |
|  | 0.5209 | 0.453 | 0.3896 |  |  |  |  |  |  |  |  |
| **LWP** | -0.63705 | -0.14666 | -0.85633 | -0.48703 |  |  |  |  |  |  |  |
|  | 0.1737 | 0.7816 | 0.0295 | 0.3272 |  |  |  |  |  |  |  |
|  |  |  |  |  |  |  |  |  |  |  |  |
|  |  |  |  |  |  |  |  |  |  |  |  |
| **2018 nonirr 5601T** | |  |  |  |  |  | **2018 nonirr A5959** | | |  |  |
|  |  |  |  |  |  |  |  |  |  |  |  |
|  | **sucrose** | **raffinose** | **stachyose** | **yield** |  |  |  | **sucrose** | **raffinose** | **stachyose** | **yield** |
| **raffinose** | -0.71451 |  |  |  |  |  | **raffinose** | 0.30968 |  |  |  |
|  | 0.1106 |  |  |  |  |  |  | 0.5503 |  |  |  |
| **stachyose** | 0.78995 | -0.83372 |  |  |  |  | **stachyose** | 0.70598 | -0.09641 |  |  |
|  | 0.0615 | 0.0392 |  |  |  |  |  | 0.117 | 0.8558 |  |  |
| **yield** | -0.05255 | -0.25503 | -0.09882 |  |  |  | **yield** | 0.35252 | -0.47242 | 0.64856 |  |
|  | 0.9213 | 0.6258 | 0.8523 |  |  |  |  | 0.4931 | 0.3441 | 0.1636 |  |
| **LWP** | 0.47565 | -0.41588 | 0.00129 | 0.44522 |  |  | **LWP** | -0.27332 | 0.32343 | -0.27488 | -0.41948 |
|  | 0.3403 | 0.4121 | 0.9981 | 0.3763 |  |  |  | 0.6002 | 0.5318 | 0.5981 | 0.4077 |
|  |  |  |  |  |  |  |  |  |  |  |  |
|  |  |  |  |  |  |  |  |  |  |  |  |
| **2018 nonirr Hutcheson** | | |  |  |  |  | **2018 nonirr N987265** | | |  |  |
|  |  |  |  |  |  |  |  |  |  |  |  |
|  | **sucrose** | **raffinose** | **stachyose** | **yield** |  |  |  | **sucrose** | **raffinose** | **stachyose** | **yield** |
| **raffinose** | 0.06005 |  |  |  |  |  | **raffinose** | -0.01673 |  |  |  |
|  | 0.91 |  |  |  |  |  |  | 0.9749 |  |  |  |
| **stachyose** | 0.35315 | 0.33283 |  |  |  |  | **stachyose** | -0.37153 | -0.65904 |  |  |
|  | 0.4923 | 0.5192 |  |  |  |  |  | 0.4683 | 0.1546 |  |  |
| **yield** | 0.44831 | 0.74127 | 0.14191 |  |  |  | **yield** | 0.00546 | 0.83559 | -0.6471 |  |
|  | 0.3726 | 0.0918 | 0.7886 |  |  |  |  | 0.9918 | 0.0383 | 0.1648 |  |
| **LWP** | -0.77774 | 0.08314 | -0.01781 | -0.21689 |  |  | **LWP** | -0.45736 | -0.27372 | 0.29661 | -0.52741 |
|  | 0.0686 | 0.8756 | 0.9733 | 0.6798 |  |  |  | 0.3618 | 0.5997 | 0.5681 | 0.2822 |
|  |  |  |  |  |  |  |  |  |  |  |  |
|  |  |  |  |  |  |  |  |  |  |  |  |
| **2018 nonirr PI471938** | | |  |  |  |  |  |  |  |  |  |
|  |  |  |  |  |  |  |  |  |  |  |  |
|  | **sucrose** | **raffinose** | **stachyose** | **yield** |  |  |  |  |  |  |  |
| **raffinose** | 0.30157 |  |  |  |  |  |  |  |  |  |  |
|  | 0.5614 |  |  |  |  |  |  |  |  |  |  |
| **stachyose** | -0.20166 | 0.7236 |  |  |  |  |  |  |  |  |  |
|  | 0.7016 | 0.104 |  |  |  |  |  |  |  |  |  |
| **yield** | 0.16056 | 0.18702 | -0.32579 |  |  |  |  |  |  |  |  |
|  | 0.7612 | 0.7227 | 0.5286 |  |  |  |  |  |  |  |  |
| **LWP** | -0.42716 | 0.67008 | 0.84167 | -0.18369 |  |  |  |  |  |  |  |
|  | 0.3982 | 0.1453 | 0.0356 | 0.7276 |  |  |  |  |  |  |  |
